# Potential of alarmin-targeted bispecific and combination therapies in airway disease

**DOI:** 10.3389/falgy.2025.1700060

**Published:** 2025-12-02

**Authors:** H. Kiyomi Komori, Hector Ortega

**Affiliations:** 1 Translational Medicine, Uniquity Bio, San Diego, CA, United States; 2 Clinical Development, Prana Therapies, San Diego, CA, United States

**Keywords:** alarmins, asthma, biologics, chronic obstructive pulmonary disease (COPD), TSLP, IL-33, IL-25, respiratory

## Abstract

Thymic stromal lymphopoietin (TSLP), IL-33, and IL-25 are epithelial-derived proinflammatory alarmin cytokines that drive inflammatory airway diseases such as asthma and chronic obstructive pulmonary disease (COPD). Targeted inhibition of these proteins has demonstrated varying degrees of efficacy in patients with asthma and COPD. As the biology of inflammatory respiratory disease is complex, combination approaches that directly inhibit multiple targets may provide deeper efficacy in a broader patient population. Here, we review the biology of alarmins and the development landscape for monotherapies and multispecific alarmin inhibitors.

## Introduction

Airway epithelium is exposed to a variety of environmental stimuli that induce the expression of the alarmin cytokines thymic stromal lymphopoietin (TSLP), IL-33, and IL-25 (1, 2). TSLP, IL-33, and IL-25 bind to TSLPR, ST2, and IL-25R, respectively, and induce proinflammatory signaling cascades that contribute to the pathogenesis of inflammatory respiratory diseases, including asthma, chronic obstructive pulmonary disease (COPD), and chronic rhinosinusitis with nasal polyps (CRSwNP) (1, 3, 4). As such, inhibition of alarmin signaling has emerged as an attractive approach to reducing inflammation and symptoms in patients with inflammatory respiratory disease. Tezepelumab, a monoclonal antibody directed against TSLP, is approved for the treatment of patients with moderate-to-severe asthma and is in development for other type 2 inflammatory diseases. Inhibition of IL-33 is being explored as a potential therapy for COPD, while IL-25 is being targeted in asthma and idiopathic pulmonary fibrosis. As upstream mediators of inflammation, inhibition of alarmin signaling may result in broader immune modulation than targeting downstream cytokines such as IL-4, IL-5, and IL-13 alone. Therapeutics that target multiple cytokines (e.g., TSLP and IL-4) may result in even broader immune modulation and provide greater efficacy in patients with heterogenous disease or incomplete response with monotherapies.

## Alarmins

### TSLP

**Figure 1:**
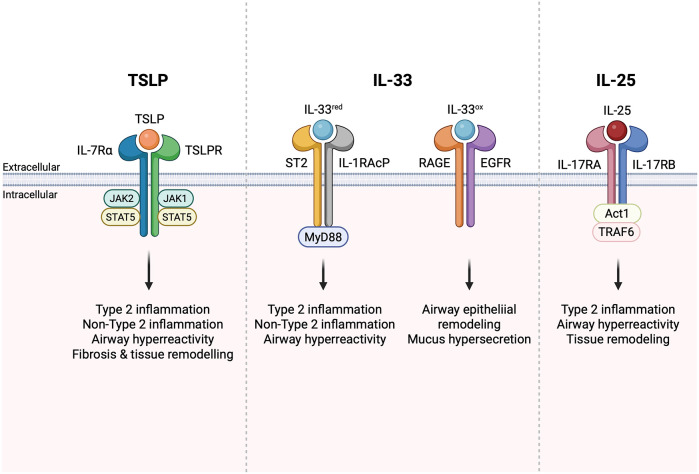
Alarmin signaling drives type 2 and non-type 2 inflammation. Figure created in BioRender.com.

TSLP is a proinflammatory alarmin that functions as a master regulator of type 2 inflammation. Barrier tissue epithelial cells are the primary expressors of TSLP, secreting the alarmin in response to danger signals, allergens, environmental antigens, and infectious agents (2). Bronchial smooth muscle cells, mast cells, and basophils also express TSLP (2, 5). TSLP binds to a heterodimeric receptor composed of the TSLP receptor (TSLPR) and the IL-7 receptor alpha chain (IL-7RA). Upon binding to TSLPR/IL-7RA, TSLP signals through Janus kinase (JAK) JAK1/JAK2 and signal transducer and activator of transcription (STAT) 5 (Figure 1) and, to a lesser extent, STAT3 and other transcription factors (2). Dendritic cells express TSLPR/IL-7RA and can be activated by TSLP to express TARC (CCL17), driving differentiation of Th2 T cells expressing IL-4, IL-5, IL-9, and IL-13 (6, 7). In addition to stimulating T-cell-driven Th2 responses, TSLP has been shown to modulate innate lymphoid cell (ILC) activation and survival (8, 9), stimulate epithelial cell proliferation (10), activate mast cells in synergy with IL-1 (11, 12), and contribute to tissue fibrosis (13) and remodeling (14, 15). Studies have shown that TSLP has two distinct isoforms, short-form TSLP (sfTSLP) and long-form TSLP (lfTSLP), which are considered to exert different roles (homeostatic and inflammatory) in several diseases (16). An approved anti-TSLP monoclonal antibody, tezepelumab, binds to lfTSLP (17), but binding to sfTSLP has not been evaluated. The anti-TSLP antibodies in clinical development discussed below have been tested against lfTSLP but not sfTSLP. Current clinical development programs studying TSLP inhibitors are not systematically characterizing these isoforms, and their clinical relevance remains unclear.

Through activation of Th2 and other inflammatory pathways, TSLP has been demonstrated to play important roles in driving disease pathogenesis in inflammatory diseases such as asthma and COPD (18–20). Single-nucleotide polymorphisms that elevate TSLP expression have been associated with increased severity and incidence of asthma (21, 22) and identified in patients with chronic rhinosinusitis (3). Serum and tissue levels of TSLP are elevated in patients with asthma and COPD compared with healthy controls (23, 24). Asthma airway hyperresponsiveness correlates with airway TSLP levels (23), and inhibition of TSLP with tezepelumab has proven to be an effective therapy in patients with asthma (25).

### IL-33

IL-33 is a member of the IL-1 family of cytokines that is expressed by barrier epithelial cells, endothelial cells, fibroblast-like cells, and airway smooth muscle cells (20). Full-length IL-33 is expressed as a proprotein that can be processed to a shorter form through inflammatory or apoptotic processing, which can either enhance or reduce activity (26). Despite its nuclear localization, IL-33 does not appear to have significant effects on gene transcription or regulation, although further studies in this area are warranted (27). Instead, nuclear localization provides regulatory control by sequestering IL-33 inside the cell to prevent constitutive secretion (27, 28). IL-33 is generally thought to be released from damaged cells as an alarmin, although a recent report has shown that IL-33 is secreted from DCs via perforin 2 (29). Mobilization of a RIPK1-caspase 8 “ripoptosome” in epithelial cells has also been identified as a mechanism for the release of IL-33. While full-length IL-33 has a biological function, ripoptosome-dependent cleavage by caspase 3/7 or by extracellular serine proteases generates a more potent “mature” form (27, 30). Interestingly, secreted IL-33 can remain bound to chromatin, which can synergistically activate receptor-mediated signaling, suggesting an important role for the chromatin-binding activity of IL-33 in modulating IL-33 signal strength (28). Consistent with constitutive IL-33 expression and release by dying cells, elevated IL-33 levels can be detected in a wide range of human diseases, including asthma, atopic dermatitis, ulcerative colitis, allergic rhinitis, and rheumatoid arthritis (31).

IL-33 is also regulated by oxidation-driven conformational changes that occur following secretion from cells, resulting in oxidized and reduced forms of IL-33 (32). Reduced IL-33 signals via a heterodimer formed by the IL-1 family receptor ST2 (IL1RL1) and the co-receptor IL-1 receptor accessory protein (IL-1RAcP) that is used by other members of the IL-1 cytokine family, while oxidized IL-33 signals through receptor for advanced glycation end products (RAGE)/epidermal growth factor receptor (EGFR) complex (Figure 1) (33). Ligation of the ST2/IL1RL1 receptor complex by IL-33 initiates MyD88-dependent inflammatory cascades that initiate non-type 2 inflammation. Signaling through RAGE/EGFR can initiate airway epithelial remodeling and is associated with mucus hypersecretion in *in vitro* COPD models (33). In a mouse model of ovalbumin-induced asthma, administration of anti-IL-33 antibodies was shown to decrease eosinophil infiltration, IgE production, and Th2 cytokine release (34), as well as airway hyperreactivity (AHR) (35). Specific targeting of ST2 [interleukin 1 receptor-like 1 (IL1RL1)], an IL-33 receptor, demonstrated similar effects in ovalbumin-challenged mice (36). In a mouse model of persistent house dust mite (HDM)-induced asthma characterized by mixed granulocytic influx in the lungs, the anti-IL-33 treatment was shown to prevent airway remodeling (37). Despite the substantial role of IL-33 in driving Th2-mediated responses and the genetic data supporting its role in airway inflammation, ST2-deficient mice are not resistant to allergic asthma (38), suggesting that inhibition of reduced IL-33 alone may not be sufficient for preventing the development of asthma. This further validates the premise that enhanced efficacy could be achieved by the inhibition of combined alarmins. The detailed analysis of allergic responses in ST2 knockout mice revealed the compensatory increase of TSLP production in response to allergen challenge (39). To test whether anti-IL-33 or anti-TSLP antibodies could attenuate inflammation, RAG1^−/−^ (recombination activating gene 1 knockout) mice were intravenously sensitized by adoptively transferred ILC2 cells from immunocompetent mice and then intranasally challenged with eosinophil extracellular traps. The study found that anti-IL-33, but not anti-TSLP, reduced IL-5 and IL-13 production, while AHR was decreased only in anti-TSLP-treated mice (40). This highlights the “complementary” role between these pathways. Taken together, these findings suggest that simultaneous targeting of multiple alarmins may provide additive or synergistic therapeutic effects for respiratory diseases.

### IL-25

IL-25, or IL-17E, is a member of the IL-17 family of cytokines that regulates type 2 immunity (41, 42). IL-25 signals via a heterodimeric receptor (IL-25R) composed of IL-17RB and IL-17RA (Figure 1), the latter of which is shared with receptors for other IL-17 family members (43). In most cells, IL-25R signaling leads to TRAF6- and Act1-dependent activation of NF-kB and AP-1 transcription factors. Expression of the IL-25 receptor is more restricted than that of ST2 and TSLPR. Some IL-17RB can be detected on myeloid cells, granulocytes, and certain T cell subsets, but expression is much higher on ILC2s, particularly in the small intestine, and on certain thymic subsets (9). Administration of anti-IL-25 antibodies in mice with ovalbumin (44) or HDM and adenoviral Smad2 overexpression-induced asthma (45) significantly decreased the Th2 immune responses and attenuated AHR and airway tissue remodeling. Clinical trials of anti-IL-25 efficacy for asthma or COPD have yet to demonstrate its role in modulating inflammation.

IL-25 has also been implicated in numerous models of allergic airway inflammation, including asthma, fibrosis, and CRSwNP (46). It is important to note, however, that generally IL-25 is not the dominant innate type 2 cytokine in the lung (47). Airway ILC2s express far more ST2 than IL-17RB (167), and while both IL-33 and IL-25 are produced by nasal epithelial cells during HDM-induced allergic rhinitis, defects in eosinophil and goblet cell counts are seen only in IL-33^−/−^ and not IL-25^−/−^ mice (48). Similarly, comparison of IL-17RB-deficient and ST2-deficient mice in models of allergic asthma has shown that IL-33 induces expansion of IL-13-producing ILC2s more potently, correlating with airway constriction (48). In asthma models, lung IL-25 levels are augmented by rhinovirus (RV) infection, and IL-25R blockade inhibits RV-induced exacerbation (49). Conversely, RV infection drives an increase in epithelial production of IL-33 and TSLP as well as increases in IL-13-secreting ILC2s in neonatal but not mature mice (5). Importantly, RV-induced disease was attenuated by anti-IL-33 treatment and in TSLPR knockout mice, suggesting a complex interplay among IL-25, IL-33, and TSLP during viral infections in the lung.

## Clinical data with alarmins

Clinical development of alarmin-targeted therapies is a growing and rapidly evolving space. To allow for inclusion of emerging data, some data summarized in this section are derived from conference presentations, corporate presentations, or press releases and have not undergone peer review. Data from such sources are informative, but should be interpreted with caution.

## Monotherapies

### TSLP

At the time of writing, there are 21 anti-TSLP monoclonals in preclinical through phase 3 clinical development. The following section focuses on those with active clinical development and available data to allow for comparisons based on preclinical or clinical attributes. Table 1 includes the additional antibodies not discussed in detail below.

**Table 1:** Monoclonal alarmin inhibitors in development for inflammatory respiratory diseases.

Therapeutic	Sponsor	Target	Ig	ROA	Indication	Current development stage	Study ID	Study status	Estimated primary completion
TSLP inhibitors									
Tezepelumab (TEZSPIRE, AMG157)	AstraZeneca/Amgen	TSLP	IgG2	SC	Asthma	Marketed	NAVIGATOR, PATHWAY	Completed	
					COPD	Phase 3	EMBARK	Recruiting	March 2029
					COPD	Phase 3	JOURNEY	Recruiting	March 2029
					CRSwNP	Phase 3	WAYPOINT	Complete	September 2024
					EoE	Phase 3	CROSSING	Active, not recruiting	July 2026
Bosakitug (ATI-045, TQC2731)	Aclaris/CTTQ	TSLP	IgG1	SC	Asthma (China)	Phase 3	NCT06829784	Recruiting	June 2027
					COPD (China)	Phase 2	NCT06707883	Recruiting	December 2026
					AD	Phase 2	NCT07011706	Recruiting	August 2026
					CRSwNP (China)	Phase 2	NCT07107256	Not yet recruiting	March 2027
GSK5748283 (AIO-001, SHR-1905)	GSK/Hengrui Pharma	TSLP		SC	Asthma	Phase 2	NCT06748053	Recruiting	July 2026
					Asthma (China)	Phase 2	NCT05593250	Active, not recruiting	April 2025
					CRSwNP (China)	Phase 2	NCT05891483	Active, not recruiting	February 2026
Solrikitug (MK-8226)	Uniquity Bio	TSLP	IgG1	SC	Asthma	Phase 2	RANIER	Recruiting	December 2025
					COPD	Phase 2	ZION	Recruiting	June 2026
					EoE	Phase 2	ALAMERE	Recruiting	April 2027
Verekitug (UPB-101, ASP7266)	Upstream	TSLPR	IgG1	SC	Asthma	Phase 2	VALIANT	Active, not recruiting	February 2026
					COPD	Phase 2	NCT06981078	Recruiting	April 2029
					CRSwNP	Phase 2	VIBRANT	Completed	July 2025
AZD8630	AstraZeneca	TSLP	FAb	Inhaled	Asthma	Phase 2	LEVANTE	Recruiting	March 2026
WIN378 (HBM9378)	Windward Bio	TSLP	IgG1	SC	Asthma	Phase 2	NCT07120503	Recruiting	July 2027
TAVO101	Tavotek	TSLP	IgG1	IV	AD	Phase 2	NCT06176040	Active, not recruiting	March 2025
CM326	Keymed	TSLP	IgG1	SC	Asthma (China)	Phase 2	CTR20230693	Completed	2025
					CRSwNP (China)	Phase 2	NCT06372678	Active, not recruiting	Not disclosed
MG-014 (MG-ZG122)	Mabgeek	TSLP		SC	Asthma (China)	Phase 2	NCT07054034	Active, not recruiting	May 2026
QX0008N	Qyuns Therapeutics	TSLP	IgG1	SC	Asthma (China)	Phase 1	CTR20231102	Recruiting	Not disclosed
GB-0895	Generate Biomedicines	TSLP			Asthma, COPD	Phase 1	NCT07116889	Recruiting	May 2027
STSA-1201	Staidson	TSLP		SC	Asthma (China)	Phase 1		Completed	
GR-2002	Genrix	TSLP	IgG1	SC	Asthma (China)	Phase 1			
A378	KLUS Pharma	TSLP			Asthma	Preclinical			
HXN-1011	Helixon	TSLP			Asthma, COPD	Preclinical			
KINE-201	Kinesciences	TSLP			Asthma, COPD	Preclinical			
Undisclosed	Scinai	TSLP			Asthma, AD	Preclinical			
SOR-104	Sorriso Pharmaceuticals	IL-7RA	sdAb	PO/inhaled		Preclinical			
IL-33 inhibitors									
Astegolimab	Roche	ST2	IgG2	SC	COPD	Phase 3	ARNASA	Recruiting	June 2025
					COPD	Phase 2	NCT05037929	Active, not recruiting	February 2025
Itepekimab	Regeneron/Sanofi	IL-33		SC	COPD	Phase 3	AERIFY-1	Active, not recruiting	April 2025
						Phase 3	AERIFY-2	Active, not recruiting	April 2025
Tozorakimab	AstraZeneca/Amgen	IL-33		SC	COPD	Phase 3	OBERON	Active, not recruiting	January 2026
						Phase 3	TITANIA	Active, not recruiting	January 2026
						Phase 3	MIRANDA	Active, not recruiting	March 2026
TQC-2938	Sino Biopharmaceutical	ST2			COPD	Phase 2	NCT06789289	Not yet recruiting	December 2027
9MW-1911	Mabwell Bioscience	ST2		IV	COPD	Phase 1/2	NCT06175351	Active, not recruiting	September2025
GSK-3862995	GSK	IL-33			COPD	Phase 1	NCT06154837	Recruiting	June 2026
Torudokimab (ZB-880)	Zura Bio	IL-33		SC	Asthma	Phase 1	NCT03343587	Completed	
FB-918	Oneness Biotech	IL-33				Preclinical			
QX-007N	Qyuns Therapeutics	IL-33				Preclinical			
IL-25 inhibitors									
XKH-001	Suzhou Kanova Biopharmaceutical	IL-25	IgG1	SC	AD	Phase 2	NCT07054736	Not yet recruiting	October 2025
SM-17	SinoMab BioScience	IL-17BR (IL-25R)	IgG4		Asthma	Phase 1			
			IgG4		AD	Phase 1	NCT07103369	Completed	
			IgG4		IPF	Preclinical			
LNR-125.38 (ABM125)	Lanier Biotherapeutics	IL-25			Asthma, CRSwNP, AD, EoE	Preclinical			
Undisclosed	Novarock Biotherapeutics	IL-25			Asthma, AD, COPD, UC	Preclinical			

AD, atopic dermatitis; COPD, chronic obstructive pulmonary disease; CRSwNP, chronic rhinosinusitis with nasal polyps; EoE, eosinophilic esophagitis; IPF, idiopathic pulmonary fibrosis; UC, ulcerative colitis.

#### Tezepelumab

##### Asthma

Tezepelumab is a fully humanized monoclonal anti-TSLP antibody developed by AstraZeneca. It is the first alarmin-targeted biologic to be approved, attaining marketing approval for the treatment of patients with asthma in 2021 (50). Tezepelumab is an IgG2 with a half-life of ∼26 days (50). Its efficacy and safety were evaluated in 52-week, randomized, double-blind, placebo (PBO) controlled phase 2 (NCT02054130/PATHWAY, *n* = 550) and phase 3 (NCT03347279/NAVIGATOR, *n* = 1,061) studies in patients with asthma (25, 51). PATHWAY was a dose-ranging study evaluating subcutaneous tezepelumab administered at 70 mg Q4W (*n* = 138), 210 mg Q4W (*n* = 137), 280 mg Q2W (*n* = 137), and PBO (*n* = 138). Based on the results of the study, 210 mg Q4W was selected as the efficacious dose to be tested in the phase 3 NAVIGATOR study. The following summary focuses on the results of the 210 mg Q4W-treated group in the PATHWAY study. As summarized in Table 2, in both clinical studies, 210 mg Q4W tezepelumab significantly reduced the annualized asthma exacerbation rate (AAER) and improved pre-bronchodilator forced expiry volume in 1 s (FEV1) and symptom scores compared with PBO. Biomarkers of type 2 inflammation were also reduced by tezepelumab treatment. Eosinophils were significantly reduced beginning at week 4, reaching a reduction of 50% by week 52 (59). Reductions in fractional exhaled nitric oxide (FeNO) were observed as early as week 4 (the earliest timepoint sampled) and with the maximal reduction in FeNO observed at week 8 and maintained through the end of treatment (51). Additionally, other inflammatory biomarkers of type 2 inflammation (IL-5, IL-13, and TARC) were reduced in the phase 2b PATHWAY study with tezepelumab (59). In both the PATHWAY and NAVIGATOR studies, tezepelumab was generally safe and well-tolerated. In the phase 3 NAVIGATOR study, 9.8% of tezepelumab-treated patients reported a serious adverse event compared with 13.7% in the PBO-treated group. Incidence of severe infections did not differ between the tezepelumab- and PBO-treated groups (8.7% each). The most common adverse events were nasopharyngitis, upper respiratory tract infection, headache, and asthma. At or after baseline, 0.8% (PATHWAY) and 4.9% (NAVIGATOR) of tezepelumab-treated patients were positive for antidrug antibodies (ADA), with only a single patient having detectable levels of neutralizing ADA in the NAVIGATOR study.

**Table 2:** Efficacy of monoclonal alarming inhibitors in asthma and COPD.

Asthma											
Therapeutic	Study	Phase	Study duration (weeks)	Patient subgroup	Dose *n*	*n* (total, treatment group)	Exacerbations (rate reduction vs. PBO)	AER (events per year)	FEV1 improvement from baseline (mean, placebo adjusted)	ACQ-6 (mean, placebo adjusted)	References
Tezepelumab	NCT02054130 PATHWAY	2	52	All comers	210 mg Q4W	412, 137	71%	Tez: 0.2	130 mL	−0.29	Corren et al., (51)
							*p* < 0.001	PBO: 0.72	*p* = 0.009	*p* = 0.039	
	NCT03347279 NAVIGATOR	3	52	All comers	210 mg Q4W	1,061, 529	56%	Tez: 0.93	130 mL	−0.33	Menzies-Gow et al. (25)
							*p* < 0.001	PBO: 2.1	*p* < 0.001	*p* < 0.001	
Itepekimab	NCT03387852	2	12	All comers	300 mg Q2W	296, 73	n/a	n/a	140 mL	ACQ-5	Weschler et al., (76)
										−0.42	
Chronic obstructive pulmonary disease											
Therapeutic	Study	Phase	Study duration (weeks)	Patient subgroup	Dose *n*	*n* (total, treatment group)	Exacerbations (rate reduction vs. PBO)	AER (rate ratio, CI)	FEV1 improvement from baseline (Mean, placebo adjusted)	SGRQ (mean, placebo adjusted)	References
Tezepelumab	NCT04039113 COURSE	2a	52	All comers	420 mg Q4W	333, 165	17%	Tez: 1.75	55 mL	−2.93	Singh et al. (52)
							*p* = 0.1	PBO: 2.11			
				BEC < 150 cells/μL	420 mg Q4W	333, 73	−19%	Tez: 2.04	51 mL	−1.62	
								PBO: 1.71			
				BEC ≥ 150–<300 cells/μL	420 mg Q4W	333, 66	34%	Tez:1.64	34 mL	−2.41	
								PBO: 2.47			
				BEC ≥ 300 cells/μL	420 mg Q4W	333, 24	46%	Tez: 1.20	146 mL	−9.53	
								PBO: 2.24			
Itepekimab	NCT03546907	2a	52	All comers	300 mg Q2W	343, 172	19%	Ite: 1.30	60 mL	n/a	Rabe et al. (53)
							*p* = 0.13	PBO: 1.61	*p* = 0.024		
				Former smokers	300 mg Q2W	343, 98	42%	Ite: 0.89	90 mL	n/a	
							*p* = 0.0061	PBO: 1.55	*p* = 0.0076		
				Current smokers	300 mg Q2W	343, 74	−9%	Ite: 1.86	20 mL	n/a	
							*p* = 0.65	PBO: 1.70	*p* = 0.54		
				BEC < 250 cells/μL	300 mg Q2W	343, 104	16%	Ite: 1.26	20 mL	n/a	
							*p* = 0.32	PBO: 1.51	*p* = 0.46		
				BEC ≥ 250 cells/μL	300 mg Q2W	343, 68	22%	Ite: 1.34	120 mL	n/a	
							*p* = 0.28	PBO: 1.71	*p* = 0.016		
	NCT04701983 AERIFY-1	3	52	Former smokers	Q2W	1,127, 375	27%^a^	n/a	n/a	n/a	Sanofi press release (54)
				Former smokers	Q4W	1,127, 377	21%^a^	n/a	n/a	n/a	
	NCT04751487 AERIFY-2	3	52	Former smokers	Q2W	1,239, 326	2%	n/a	n/a	n/a	Sanofi press release (54)
				Former smokers	Q4W	1,237, 324	12%	n/a	n/a	n/a	
Tozorakimab	NCT04631016 FRONTIER-4	2a	12	All comers	600 mg Q4W	135, 67	n/a	n/a	24 mL	n.s.	Singh et al. (55)
									*p* = 0.216		
				Former smokers	600 mg Q4W	136, 43	n/a	n/a	66 mL	n/a	
									*p* = 0.098		
				Current smokers	600 mg Q4W	136, 24	n/a	n/a	57 mL	n/a	
									*p* = 0.177		
				BEC < 150 cells/μL	600 mg Q4W	136, 37	n/a	n/a	−3 mL	n/a	
									*p* = 0.474		
				BEC ≥ 150 cells/μL	600 mg Q4W	136, 30	n/a	n/a	155 mL	n/a	
									*p* = 0.003		
Astegolimab	NCT03615040 COPD-ST2OP	2a	44	All comers	490 mg Q4W	81, 42	22%	Ast: 2.18	n/a	−3.3	Yousuf et al. (56)
							*p* = 0.19	PBO: 2.81		*p* = 0.039	
				Former smokers	490 mg Q4W	81, n/a	20%	n/a	n/a		
				Current smokers	490 mg Q4W	81, n/a	22%	n/a	n/a		
				BEC ≤ 170 cells/μL	490 mg Q4W	81, 31	31%	n/a	n/a	−1.26	
				BEC > 170 cells/μL	490 mg Q4W	81, 11	17%	n/a	n/a	−5.2	
	NCT05037929 ALIENTO	3	52	All comers	Q2W	1,301, n/a	15.4%^a^	n/a	n/a	n/a	Genentech press release (57)
	NCT05595642 ARNASA	3	52	All comers	Q2W	1,375, n/a	14.5%	n/a	n/a	n/a	Genentech press release (57)
Chronic rhinosinusitis with nasal polyps											
Therapeutic	Study	Phase	Study duration (weeks)	Patient subgroup	Dose, *n*	*n* (total, treatment group)	Total nasal polyp score (mean, placebo adjusted)		Nasal congestion score (mean, placebo adjusted)	SNOT-22 total score (mean, placebo adjusted)	References
Tezepelumab	NCT04851964 WAYPOINT	3	52	All comers	210 mg Q4W	408, 203	−2.07		−1.03	−27.26	Lipworth et al. (4)
							*p* < 0.001		*p* < 0.001	*p* < 0.001	
Verekitug	NCT06164704 VIBRANT	2	24	All comers	100 mg Q12W	80, 40	−1.8		−0.8	n/a	Upstream press release (58)
							*p* < 0.0001		*p* = 0.0003		

n/a, data not available; n.s., not statistically significant; AER, annualized exacerbation rates; BEC, blood eosinophil count.

a Statistically significant, *p*-value not reported.

##### COPD

Tezepelumab is being evaluated for the treatment of patients with moderate-to-severe COPD. In a double-blind, randomized, PBO-controlled phase 2a study (NCT04039113/COURSE, *n* = 333), moderate-to-very severe COPD patients on triple inhaled maintenance therapy were treated subcutaneously with 420 mg Q4W tezepelumab or PBO for 52 weeks (52). The primary endpoint evaluated was the annualized rate of moderate or severe COPD exacerbations. The data summarized in Table 2 demonstrate that although evaluation in the total population did not demonstrate meaningful differences compared with placebo; however, pre-specified assessment in the population of patients with baseline eosinophil counts ≥150 cells/mL showed that tezepelumab may reduce exacerbations and symptoms and improve FEV1 in COPD patients with type 2 high inflammation defined by blood eosinophil count ≥150 cells/mL. Similar results were observed when evaluating patients with FeNO ≥25 ppb during screening.

##### CRSwNP

Tezepelumab was evaluated in a phase 3, double-blind, randomized, placebo-controlled study in patients with CRSwNP (WAYPOINT, NCT04851964) (4). This study evaluated 210 mg tezepelumab Q4W compared with placebo over 52 weeks with co-primary endpoints of change from baseline in total nasal polyp score and mean nasal congestion score. Tezepelumab-treated patients had significant improvements in both the total nasal polyp score (mean difference vs. PBO −2.07; 95% CI −2.39 to −1.74, *p* < 0.001) and mean nasal congestion score (mean difference −1.03; 95% CI −1.20 to −0.86, *p* < 0.001). Tezepelumab also demonstrated significant improvements in the loss-of-smell score, 22-item sino-nasal outcome test (SNOT-22) total score, total symptom score, and reduced surgery for nasal polyps and systemic glucocorticoid use compared with placebo. Tezepelumab was found to be generally safe and well-tolerated in patients with CRSwNP. The most frequently reported adverse events were coronavirus disease 2019, nasopharyngitis, upper respiratory tract infection, headache, epistaxis, and worsening of CRSwNP (which was greater in the PBO-treated group than that in the tezepelumab-treated group, 22.9% vs. 5.4%, respectively).

In addition to respiratory programs, tezepelumab has also been evaluated for the treatment of patients with eosinophilic esophagitis (CROSSING, NCT005583227).

#### GSK5784283

GSK5784283 (SHR-1905) is a humanized anti-TSLP monoclonal antibody modified for extended half-life (60) currently in phase 2 development in asthma (NCT06748053). Phase 1 data evaluating GSK5784283 in healthy volunteers and asthma patients were presented at the European Respiratory Society (ERS) 2024 meeting (61). In this phase 1 single ascending dose (SAD) study (NCT04800263), doses ranging from 50 to 600 mg were evaluated in healthy volunteers, 200 mg in mild asthma patients, and 100 and 600 mg in moderate-to-severe asthma patients (61). GSK5784283 was generally safe and well-tolerated, with no serious adverse events occurring. The most common treatment-emergent adverse events (TEAEs, >5%) in healthy volunteers exposed to GSK5784283 (*n* = 40) were headache, COVID-19, back pain, contact dermatitis, upper respiratory tract infection, increased troponin 1, and immunization reaction (60). The most common TEAEs (≥2 subjects) were increased alanine aminotransferase and increased blood creatinine (61). The mean half-life of GSK5784283 across 50–600 mg in healthy volunteers was ∼80 days (60), supporting extended dosing regimens in future clinical studies. The impact of GSK5784283 on FEV1 and type 2 inflammatory biomarkers (eosinophils, FeNO, TARC, and eotaxin-3) was evaluated in the moderate-to-severe asthma cohort. Both the 100 and 600 mg GSK5784283 groups demonstrated improvements in FEV1 (371 and 394 mL, respectively) while the PBO-treated group showed no improvement over baseline. Dose-dependent responses were observed with the 600 mg dose arm, demonstrating larger reductions in eosinophils, TARC, and eotaxin-3 compared with the 100 mg GSK5784283- and PBO-treated groups. Reductions in FeNO were similar between the two dose arms (100 mg −38.2%, 600 mg −31.6%, PBO −8% and 7%) (61).

#### Verekitug

Verekitug (UPB-101, ASP7266) is a human anti-TSLPR monoclonal antibody currently in phase 2 development for the treatment of patients with asthma (NCT06196879), chronic rhinosinusitis with nasal polyps (VIBRANT, NCT06164704), and COPD (NCT06981078). It has been shown to be ∼6× more potent than a benchmark anti-TSLP antibody at inhibiting TSLP-induced proliferation of Ba/F3 cells expressing human TSLPR and 4× more potent than anti-TSLP at inhibiting TSLP-induced TARC expression by human peripheral blood mononuclear cells (PBMC) (62). Verekitug has been evaluated in a phase 1 multiple ascending dose (MAD) study in patients with mild-to-moderate asthma (NCT05448651). Data from this study were presented at the American Thoracic Society (ATS) 2024 (63). In this phase 1 study, subcutaneous verekitug was evaluated at 25 mg (single dose, *n* = 6), 100 mg Q4W (*n* = 6), 200 mg Q4W (*n* = 6), and 300 mg Q12W (*n* = 6) vs. placebo (*n* = 8). Participants were required to have either blood eosinophils ≥200 cells/mL or blood eosinophils ≥150 cells/mL and FeNO > 25 ppb. Verekitug was found to be generally safe and well-tolerated. No serious TEAEs were reported, and the most common TEAE reported during the study was headache [8/32 (25%)] (63). In this study, verekitug demonstrated linear pharmacokinetic elimination with a half-life of ∼20 days. Antidrug antibodies were detected in 5/24 (21%) of participants with no dose relation and no discernible impact on the pharmacodynamics (PD) profile. Receptor occupancy was evaluated by flow cytometry of peripheral monocytes. All doses demonstrated near complete receptor occupancy at week 2 (the earliest timepoint evaluated). The 25 mg single-dose group maintained receptor occupancy >75% through week 12 and then recovered to baseline by week 20. Receptor occupancy >75% was maintained to week 28 for the 100 mg Q4W group, week 24 for the 200 mg Q4W group, and to week 32 (end of study) for the 300 mg Q12W group. In this cohort of patients with mild-to-moderate asthma with a type 2 phenotype, verekitug reduced eosinophils (week 32 −52% 100 mg Q4W, −50% 200 mg Q4W, −21% 300 mg Q12W, placebo ∼25%) and FeNO (week 32 −49% 100 mg Q4W, −38% 200 mg Q4W, −45% 300 mg Q12W). No changes in lung function or asthma symptoms were observed.

Verekitug was evaluated in a phase 2, randomized, double-blind, placebo-controlled study in patients with CRSwNP (VIBRANT, NCT06164704). This study evaluated 100 mg verekitug Q12W compared with placebo over 24 weeks with a primary endpoint of change from baseline in nasal polyp score. Topline data recently released by the sponsor press release indicate that verekitug-treated patients demonstrated significant improvement in nasal polyp score (mean difference vs. PBO −1.8; 95% CI −2.51 to −1.03, *p* < 0.0001) (58). They also demonstrated significant improvements in nasal congestion score (mean difference vs. PBO −0.8; 95% CI −1.17 to −0.37 L *p* = 0.0003). Significant improvements in Lund–Mackay, total symptom, and difficulty with smell scores were also achieved in verekitug-treated patients. Verekitug was found to be generally safe and well-tolerated in patients with CRSwNP. The most frequently reported adverse events were upper respiratory tract infection, sinusitis, nasopharyngitis, and headache. Adverse events were more frequent in the placebo-treated group than the verekitug-treated group.

#### Bosakitug

Bosakitug (ATI-045, TQC2731) is a humanized anti-TSLP monoclonal antibody currently in development for atopic dermatitis (NCT07011706), asthma (NCT06829784), and COPD (NCT06707883). Sponsor documents demonstrate that bosakitug binds to TSLP with a binding affinity similar to a benchmark anti-TSLP antibody, but has a longer off-rate, allowing for the potential for extended dosing durations (64). Data from a phase 1 study were presented at ERS 2024 (65). In this phase 1 single ascending dose study in healthy Chinese volunteers evaluating subcutaneous doses ranging from 12 to 630 mg, bosakitug was found to be generally safe and well-tolerated. No drug-related serious adverse events were observed, and the incidence of adverse events was not dose-dependent. The half-life of bosakitug ranged from 17.2 to 25 days (65).

#### CM326

CM326 is a humanized anti-TSLP monoclonal antibody in phase 2 development in China for chronic rhinosinusitis with nasal polyps (NCT06372678). Preclinical *in vitro* assays demonstrate that CM326 is more potent than a benchmark anti-TSLP antibody at inhibiting TSLP-induced cell proliferation (5×) and PBMC TARC inhibition (4×) (66). Single ascending dose (NCT04842201) and MAD (NCT05171348) phase 1 clinical studies in healthy volunteers have been completed for CM326. In these studies, CM326 was found to be generally safe and well-tolerated (66). The SAD study evaluated doses ranging from 22 to 880 mg CM326 and demonstrated a half-life ranging from 20.1 to 24.5 days. Antidrug antibodies were detected in 2/58 (3%) subjects in the SAD study and 3/44 (7%) of subjects in the MAD study.

#### TAVO101

TAVO101 is a humanized anti-TSLP monoclonal antibody modified for extended half-life (LS mutation) in phase 2 development for atopic dermatitis (NCT06176040) and plans for development in asthma, COPD, and rhinitis. It binds to TSLP with similar affinity as a benchmark anti-TSLP antibody, but binds to a different epitope, and is 12× more potent at inhibiting TSLP-induced STAT5 activation. In an acute OVA model of asthma in TSLP/TSLPR humanized mice, TAVO101 reduces total serum IgE, lung TARC, IL-13, and eosinophil accumulation similarly to a benchmark anti-TSLP (67). In a phase 1 SAD study (NCT05298046) in healthy volunteers evaluating intravenous doses ranging from 0.01 to 10.0 mg/kg, TAVO101 was found to be generally safe and well-tolerated with no serious adverse events reported (68). The median half-life of TAVO101 was found to be 67 days, and no antidrug antibodies were detected in the 27 volunteers dosed with TAVO101.

#### Solrikitug

Solrikitug is a humanized anti-TSLP monoclonal antibody in phase 2 development for asthma (NCT06496607), COPD (NCT06496620), and EoE (NCT06598462). *In vitro* pharmacology data were presented at ATS 2025 (69). Solrikitug binds a different epitope on TSLP than commercial tezepelumab, and surrogates for other anti-TSLP antibodies in development, blocking the binding of TSLP to IL-7RA (site II) rather than blocking the binding of TSLP to TSLPR (site I). *In vitro* characterization demonstrates that solrikitug is ∼10× more potent than commercial tezepelumab at inhibiting TSLP-induced biologic activity (human PBMC TARC expression, receptor dimerization, STAT5 activation) (69). Enhanced functional potency may allow for extended dosing durations without Fc half-life modification.

#### AZD8630

AZD8630/AMG104 is an anti-TSLP fragment antibody (FAb) formulated for dry powder inhaler delivery. It is currently in phase 2 development for the treatment of patients with uncontrolled asthma at risk of exacerbations (NCT06529419/LEVANTE). Phase 1 study (NCT05110976) data were presented at ATS 2024 (70) and ERS 2024 (71). In this study, AZD8630 was evaluated at doses ranging from 0.2 to 16 mg inhaled QD in healthy volunteers (part A) and patients with moderate-to-severe asthma (part B). In part B, 77 asthma patients with elevated FeNO and treatment with ICS/LABA were randomized to 0.4, 2, or 8 mg AZD8360 or placebo QD for 28 days. Daily inhaled 8 mg AZD8630 resulted in a significant reduction in FeNO compared with PBO (23% reduction vs. PBO, *p* = 0.037). FeNO reductions were observed at day 7 and sustained through day 28 (end of treatment). AZD8630 was found to be generally safe and well-tolerated with no serious adverse events in either part A or part B. Pharmacokinetic assessment demonstrated that AZD8630 was steadily absorbed following inhalation with two- to threefold accumulation in serum at steady state and a mean terminal half-life of 21–37 h across cohorts. Low immunogenicity was observed, with 3 (2.4%) of treated individuals developing ADA.

#### Ecleralimab

Ecleralimab is an anti-TSLP IgG1λ Fab (72) that was in development for asthma. A proof-of-concept, randomized, double-blind, placebo-controlled phase 2a study (NCT03138811) evaluated the safety, tolerability, PK, and PD of multiple ecleralimab doses in patients with mild atopic asthma before and after inhaled allergen challenge (73). The study was conducted over a 12-week treatment period with allergen challenge trials conducted on days 41–43 and 83–85. Ecleralimab 4 mg QD was delivered via dry powder inhaler. At day 84, ecleralimab significantly attenuated late asthmatic response AUC_3–7 h_ compared with PBO [4.2% vs. 11.38%, Δ −7.18% (90% CI −11.92% to −2.44%), *p* = 0.008]. Ecleralimab induced a numerical attenuation of early asthmatic responses at days 42 and 48 that was not statistically significantly different than PBO. Ecleralimab significantly reduced sputum eosinophils compared with placebo at 7 h (62%) and 24 h (52%) post-allergen challenge. No difference in pre-allergen challenge eosinophils was observed between ecleralimab and PBO-treated patients. Ecleralimab significantly reduced FeNO compared with PBO by day 17 (−27% vs. −7%, *p* = 0.03); this reduction was maintained over the treatment period. However, there was no difference in FeNO between treatment groups on days 43 and 85 (24 h post-allergen). In this study, ecleralimab was found to be generally safe and well-tolerated with AEs comparable between treatment groups. The most frequent AEs reported were headache (25%), nasopharyngitis (17.9%), and oropharyngeal pain (17.9%). No severe or serious AEs were reported. A phase 2 study evaluating the efficacy and safety of ecleralimab in patients with severe uncontrolled asthma (NCT04410523) was terminated early due to sponsor decision.

#### GB-0895

GB-0895 is an anti-TSLP monoclonal antibody engineered to have an extended half-life and is currently in phase 1 development in patients with asthma and COPD (NCT07116889). Preclinical *in vitro* studies presented at ERS 2024 show that GB-0895 is more potent than a benchmark anti-TSLP antibody at inhibiting TSLP-induced TARC expression in PBMCs (74). In a mouse model of asthma, GB-0895 reduced eosinophil infiltration and cytokine expression in the lung.

Additional anti-TSLP antibodies are in preclinical through phase 2 development (Table 1). HXN-1011 is a biparatopic antibody targeting both the TSLPR and IL-7RA binding sites on TSLP (75). Preclinical data presented at ATS 2025 demonstrated that HXN-1011 induced greater inhibition of inflammatory cytokine expression compared with a benchmark anti-TSLP antibody in a mouse model of asthma (75).

### IL-33

#### Itepekimab

Itepekimab is a fully humanized anti-IL-33 monoclonal antibody currently being developed for the treatment of COPD. Itepekimab binds to the reduced form of IL-33 with subnanomolar affinity (37). In a randomized, double-blind, PBO-controlled phase 2a study (NCT03546907), 300 mg Q2W itepekimab (*n* = 172) was evaluated vs. PBO (*n* = 171) in patients with moderate-to-severe COPD receiving dual or triple inhaled therapy (53). This study enrolled both current and former smokers. In the all-comers analysis, the primary endpoint of the annualized rate of moderate-to-severe COPD exacerbations was not met (Table 2). The key secondary endpoint of change in pre-bronchodilator FEV1 from baseline to weeks 16–24 was met, with itepekimab demonstrating improvements in FEV1 compared with placebo. When assessed in the subgroup of patients who were former smokers, itepekimab demonstrated nominally significant reductions in acute COPD exacerbations compared with PBO and improvement in FEV1. Two randomized, double-blind, PBO-controlled phase 3 studies (NCT04701983/AERIFY-1, NCT04751487/AERIFY-2) evaluating itepekimab dosed Q2W and Q4W in patients with COPD were recently completed, and topline data shared by sponsor press release (54). AERIFY-1 (*n* = 1,127) evaluated itepekimab in former smokers, and AERIFY-2 evaluated itepekimab in both former and current smokers. The AERIFY-1 study met the primary endpoint of exacerbation reduction in former smokers at week 52 (Q2W 27%, Q4W 21%), whereas the AERIFY-2 study did not meet the primary endpoint of exacerbation reduction in former smokers at week 52 (Q2W 2%, Q4W 12%).

In a randomized, double-blind, PBO-controlled phase 2 study (NCT03387852), 300 mg Q2W itepekimab (*n* = 73) was evaluated compared with 300 mg Q2W dupilumab (*n* = 75), 300 mg Q2W dupilumab plus 300 mg Q2W itepekimab (*n* = 74), and placebo (*n* = 74) in adults with moderate-to-severe asthma receiving LABAs (76). The primary endpoint, event indicating loss of asthma control at week 12, was lower in all active treatment arms compared with placebo (itepekimab 22%, dupilumab 19%, itepekimab + dupilumab 27%, PBO 41%). Correspondingly, the odds ratios compared with PBO were 0.42 (95% CI 0.2–0.88; *p* = 0.020) for itepekimab, 0.52 (95% CI 0.26–1.06; *p* = 0.07) for the combo treatment, and 0.33 (95% CI 0.15–0.7) for dupilumab. Increases in FEV1 were observed with itepekimab and dupilumab monotherapy but not with combination treatment [least squares mean (LSM) difference vs. PBO (95% CI): 0.14 L (0.01–0.27), 0.16 L (0.03–0.29), 0.1 L (−0.03–0.23), respectively]. Improvements in symptoms assessed by ACQ-5 were observed for all treatment groups compared with placebo [LSM difference vs. PBO (95% CI): itepekimab −0.42 (−0.73 to −0.012), dupilumab −0.46 (−0.76 to −0.15), combo −0.32 (−0.63 to −0.01)]. All treatments were generally safe and well-tolerated. Adverse events occurred at similar rates across all treatment groups, including the combination itepekimab + dupilumab group. While improved efficacy was not demonstrated in this study, there was no increased safety risk observed with the combination of IL-33 and IL-4/13 inhibition during the course of the study. Further development of itepekimab in patients with asthma is not being pursued.

#### Tozorakimab

Tozorakimab is a human anti-IL-33 monoclonal antibody currently in development for the treatment of COPD. It was designed to be a highly potent antibody that binds the reduced form of IL-33 with subpicomolar affinity and also the oxidized form of IL-33, thereby inhibiting signaling through both the ST2 and RAGE/EGFR pathways (33). In a randomized, double-blind, placebo-controlled phase 2a study (NCT04631016/FRONTIER-4), 600 mg Q4W tozorakimab (*n* = 67) was evaluated vs. placebo (*n* = 68) in patients with moderate-to-severe COPD with chronic bronchitis receiving dual or triple inhaled therapy (55). This study enrolled both current and former smokers, and the primary endpoint was the change in pre-bronchodilator FEV1 from baseline to week 12. While the primary endpoint was not met, tozorakimab demonstrated a numerically greater increase in FEV1 than placebo [least squares mean 24 mL (80% CI −15 to 63), *p* = 0.216]. In a pre-defined subgroup of patients with baseline blood eosinophil counts ≥50 cells/mL, tozorakimab demonstrated improvements in pre-BD FEV1 compared with placebo [LSM 82 mL (80% CI 26–138), *p* = 0.031]. The effects of tozorakimab on FEV1 at week 12 in current and former smokers were similar. Randomized, double-blind, placebo-controlled phase 3 studies evaluating tozorakimab vs. placebo are ongoing (NCT06040086/MIRANDA, NCT05166889/OBERON, NCT05158387/TITANIA).

#### Astegolimab

Astegolimab is a fully human anti-ST2 monoclonal antibody currently in development for the treatment of COPD. It inhibits reduced IL-33 signaling by inhibiting the binding of reduced IL-33 to ST2. A randomized, double-blind, placebo-controlled phase 2a study (NCT03615040/COPD-ST2OP) evaluated 490 mg astegolimab Q4W (*n* = 42) vs. placebo (*n* = 39) in patients with moderate-to-very severe COPD receiving dual or triple inhaled therapy (56). The primary endpoint, exacerbation rate at 48 weeks, was not statistically significant in the all-comers population (Table 2). Numerically greater reductions in exacerbation rate were observed in patients with eosinophil counts of ≤170 cells/mL. Smoking status did not impact response to astegolimab. Astegolimab was generally well-tolerated, and adverse and serious adverse events were similar between astegolimab and placebo-treated patients. Data from two randomized, double-blind, placebo-controlled studies (ALIENTO, NCT05037929 phase 2b; ARNASA, NCT05595642 phase 3) were recently completed, and topline data were shared by sponsor press release (57). Both studies evaluated astegolimab dosed Q2W and Q4W in patients with COPD on top of standard of care maintenance therapy (ICS/LABA, LAMA/LABA, or ICS/LAMA/LABA) and enrolled both current and former smokers. Astegolimab Q2W treatment resulted in a statistically significant reduction in exacerbations compared with placebo in the phase 2 ALIENTO study, but not in the phase 3 ARNASA study (Table 2). The safety profile of astegolimab in these studies was consistent with previously reported data.

#### TQC2938

TQC2938 is a human anti-ST2 monoclonal antibody in development for the treatment of COPD. As the phase 1 study evaluated safety, tolerability, and pharmacokinetics (PK) of TQC2938 in healthy Chinese volunteers and evaluated single SC doses (52.5–1,260 mg) and a single IV dose (210 mg) of TQC2938 compared with placebo (77). It was found to be generally well-tolerated at all doses administered. No treatment-related serious adverse events were reported. TQC2938 demonstrated linear pharmacokinetics with a half-life of 9.77–19.9 days. It is currently being evaluated in a phase 2 study in patients with COPD (NCT06789289).

Collectively, the clinical data from studies evaluating the efficacy of IL-33 inhibitors in patients with COPD have demonstrated modest efficacy in the all-comers patient populations. Phase 2 data with itepekimab indicate potentially greater efficacy in former smokers compared with current smokers. But more recent data in phase 3 produced inconsistent results across the AERIFY 1 and 2 studies. Of note, smoking status in early studies did not have an impact on efficacy in either tozorakimab or astegolimab phase 2 studies. Further data from the phase 3 studies with IL-33 inhibitors evaluating responses by smoking status and eosinophil counts may identify the most appropriate patient population for the mechanism. Data from the tozorakimab phase 3 program may also provide further insight into the importance of inhibiting both the reduced and oxidized forms of IL-33 compared with only blocking reduced IL-33 signaling.

### IL-25

#### XKH001

XKH001 is a recombinant, humanized, anti-IL-25 monoclonal antibody currently under phase 2 clinical development for the treatment of patients with atopic dermatitis. It has been evaluated in a phase 1 SAD and MAD studies in healthy Chinese volunteers (NCT05991661) evaluating doses from 100 to 600 mg dosed Q4W (78). In this study, XKH001 was well-tolerated. The mean half-life was 22–25 days with no ADA reported. Volunteers receiving the 600 mg dose had a greater decrease in IgE than placebo (XKH001 −78.92 ng/mL, PBO −8.6 ng/mL) at day 85.

#### SM17

SM17 is a humanized anti-IL-17RB IgG4 monoclonal that inhibits IL-25 signaling. In the HDM mouse model of asthma, SM17 reduced lung pathology score, eosinophil infiltration, and inflammatory cytokine (IL-4, IL-5, IL-13) secretion to levels comparable to dexamethasone-treated mice (79). Additionally, it reduced collagen deposition to a greater degree than dexamethasone. A phase 1 clinical study (NCT05332834) evaluated the safety, tolerability, PK, and PD of single (2–1,200 mg IV) and multiple ascending doses (100, 400, 600 mg IV Q2W) of SM17 in healthy volunteers (79). SM17 was found to be generally safe and well-tolerated. In the multiple ascending dose phase of the study, the most frequently reported AE was headache (50% in both SM17 and placebo). Changes in appetite/satiety were reported in 2/18 (21%) of SM17-treated volunteers and no placebo-treated volunteers. The half-life of SM17 in the MAD portion of the study was 10.5–15 days.

## Alarmin multispecific antibodies

**Figure 2:**
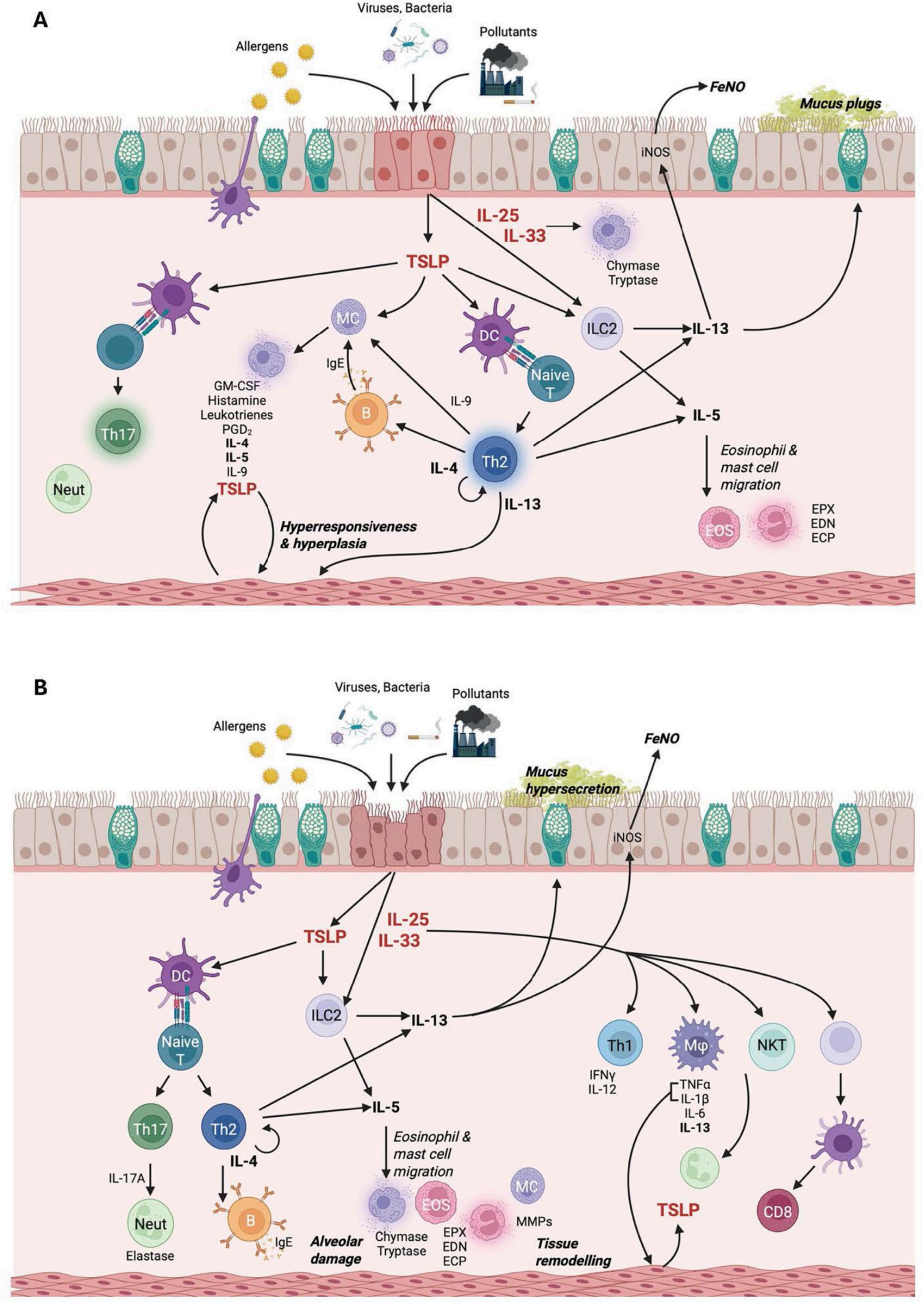
Molecular pathogenesis of **(A)** asthma and **(B)** COPD. Figure created in BioRender.com.

While monoclonal antibodies targeting single antigens (e.g., IL-4R, IL-5, TSLP, and IgE) have proven clinically effective in treating patients with respiratory disease, not all patients respond to therapy. Labels for therapies targeting IL-4R and IL-5 are limited to patients with an “eosinophilic phenotype” (80–82). Immune signaling pathways underlying disease pathogenesis are complex, with multiple immune signaling pathways contributing to different aspects of disease (Figure 2). While some patients have eosinophil-predominant disease, others have mixed phenotypes with contributions from both type 2 and non-type 2 inflammation. Direct inhibition of multiple targets through multispecific antibodies or combination therapies may provide enhanced efficacy in a broader patient population. Development of multispecific therapies for the treatment of respiratory disease is underway, with several investigational products in phase 1 and phase 2 clinical development (Table 3). While direct targeting of multiple inflammatory pathways may provide better efficacy than monotherapies, there is a risk of increased adverse events with increased immune suppression. Selection of the combination targets must be made with caution to ensure that the level of immune inhibition is sufficient to dampen disease, but not increase risk of serious and opportunistic infections. Monotherapy treatment with anti-alarmin therapies to date has been generally safe and well-tolerated, with no serious or opportunistic infections reported. Many of the bispecific/combination therapies discussed here are pairing targeting of an alarmin with IL-4/IL-13 signaling. Dupilumab (anti-IL-4RA) inhibits both IL-4 and IL-13 signaling and has proven to be safe and well-tolerated in patients with type 2 disease (80). As discussed above, a combination of IL-33 inhibition with IL-4RA inhibition did not result in increased safety risk (76). Pairing of two mechanisms with well-validated efficacy and safety profiles provides an opportunity for both enhanced efficacy and tolerable safety profiles. Safety profiles of multispecific therapies in development must be rigorously evaluated to ensure that there are no detrimental additive effects of directly inhibiting multiple immune pathways.

**Table 3:** Multispecific alarmin inhibitors in development for inflammatory respiratory disease. Figures created in BioRender.com.

Therapeutic	Sponsor	Targets	Structure	Half-life ext?	ROA	Indication	Current development stage	Study ID	Study status	Estimated primary completion
Lunsekimig	Sanofi	TSLP, IL-13	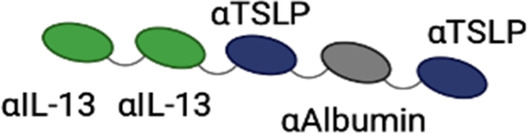	No	SC	Asthma (moderate-to-severe)	Phase 2	NCT06102005	Active, not recruiting	March 2026
					SC	Asthma (high risk)	Phase 2	NCT06676319	Recruiting	October 2027
					SC	CRSwNP	Phase 2	NCT06454240	Recruiting	January 2027
PF-07275315	Pfizer	TSLP, IL-4, IL-13	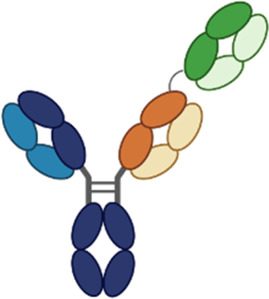	No	SC	Asthma (moderate-to-severe)	Phase 2	NCT06977581	Recruiting	March 2027
CM512	Keymed, Belenos	TSLP, IL-13			SC	Asthma (moderate-to-severe)		NCT07011524	Not yet recruiting	July 2027
					SC	COPD	Phase 2	NCT06980142	Not yet recruiting	March 2028
					SC	CRSwNP	Phase 2	NCT06930612	Not yet recruiting	September 2026
IBI3002	Innovent	TSLP, IL-4RA	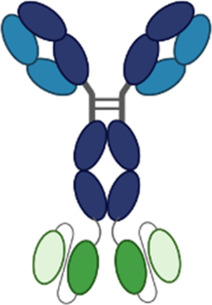		IV, SC	Asthma	Phase 1b	NCT06947408	Recruiting	April 2026
HB0056	Huabo	TSLP, IL-11	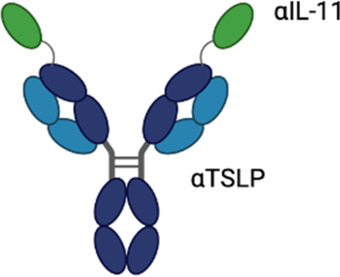				Phase 1	NCT06612970	Recruiting	June 2025
CDX-622	Celldex	TSLP, SCF	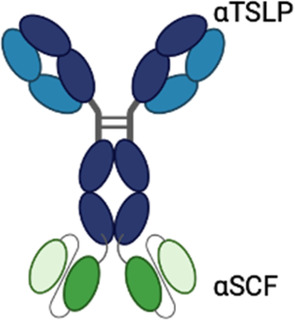				Phase 1	NCT06650761	Recruiting	January 2026
APG333	Apogee	TSLP, combo					Phase 1	ACTRN12624001358538	Active, not recruiting	December 2025
ATI-052	Aclaris	TSLP, IL-4RA	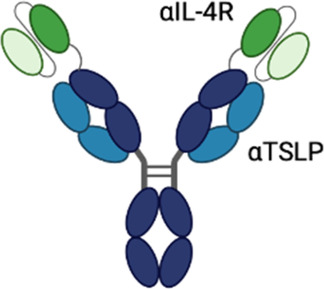				Phase 1			
ZW1528	Zymeworks	IL-33, IL-4RA	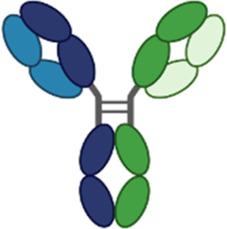				Preclinical			
HXN-1012	Helixon	TSLP, IL-13					Preclinical			
HXN-1013	Helixon	TSLP, IL-33					Preclinical			
PX-128	Johnson & Johnson	TSLP, IL-13					Preclinical			
BD9	Teva	TSLP, IL-13					Preclinical			
Undisclosed	Novarock Bio	IL-25, IL-4RA					Preclinical			

### Lunsekimig

Lunsekimig is a bispecific NANOBODY® that blocks both TSLP and IL-13 signaling (83) in development for asthma and COPD. It consists of five fragments of heavy-chain-only antibodies that are naturally occurring in camelids—two binding TSLP, two binding IL-13, and one binding human serum albumin to support FcRN-mediated recycling and extend the half-life. Unlike full IgG antibodies, heavy-chain-only antibodies cannot bind to FcRN in endosomes, leading to rapid degradation of heavy-chain-only antibodies. The addition of an FcRN-binding domain aids with protein recycling, thereby extending the half-life of the heavy-chain-only antibody (84). In *in vitro* assays, lunsekimig reduced TLSP and IL-13-induced signaling in human PBMC. In a phase 1 healthy volunteer study (EudraCT: 2021-000356-19), single ascending (10–400 mg IV, 400 mg SC) and multiple ascending (100 and 200 mg SC) doses of lunsekimig were evaluated for safety, tolerability, pharmacokinetics, and pharmacodynamics (85). Lunsekimig was well-tolerated in healthy participants, with the most frequently reported TEAEs being COVID-19, nasopharyngitis, and headache. TSLP and IL-13 levels were evaluated throughout the study. Total TSLP increased in all lunsekimig-treated groups without a clear dose-response relationship, suggesting that maximal TSLP binding was achieved at both doses evaluated. Target engagement for IL-13 was also demonstrated with IL-13 levels increasing above the assay LLOQ for most lunsekimig-treated participants. The increase in detectable circulating TSLP and IL-13 is likely a reflection of lunsekimig-bound cytokine remaining in circulation longer than free cytokine. The mean half-life of lunsekimig was ∼10 days across all IV and SC doses evaluated. Treatment-emergent ADAs were detected in 4/36 (11%) of SAD participants and 7/16 (43.8%) in the MAD portion with no apparent impact on safety, PK, or PD.

Lunsekimig has also been evaluated in a phase 1b, single-dose, double-blind, proof-of-mechanism study in patients with mild-to-moderate asthma with elevated FeNO (≥25 ppb) (NCT05366764) (85). This study evaluated the safety, tolerability, PK, PD, and immunogenicity of 400 mg SC lunsekimig (*n* = 24) compared with placebo (*n* = 12). Lunsekimig was generally well-tolerated, with the most common TEAEs for lunsekimig and placebo being nasopharyngitis (29.2% vs. 33.3%), headache (29.2% vs. 33.3%), and injection site reactions (20.8% vs. 0%). The T_1/2_ of lunsekimig was ∼10.8 days, similar to the half-life reported in the healthy volunteer study. Lunsekimig significantly reduced FeNO compared with placebo at day 8 (mean difference −33.03, 90% CI −46.31 to −19.75, *p* = 0.0001) and day 29 (−40.91 ppb, 90% CI −55.43 to −26.39, *p* < 0.0001) and maintained through day 57. Normalized FeNO levels (<25 ppb) were achieved in nine (37.5%) of lunsekimig-treated patients compared with one (8.3%) of placebo-treated patients at day 29. Lunsekimig treatment significantly reduced eosinophils and biomarkers downstream of TSLP and IL-13 at day 29. Rapid improvements in FEV1 were observed in lunsekimig-treated patients compared with placebo (mean difference 348 mL, 90% CI 147–548 mL, *p* = 0.003) at day 8. Lunsekimig is currently being evaluated in phase 2 clinical studies in patients with moderate-to-severe asthma (NCT06102005/AIRCULES), high-risk asthma (NCT06676319/AIRLYMPUS), CRWsNP (NCT06454240), and atopic dermatitis (NCT06790121) with plans to also evaluate lunsekimig in COPD.

### PF-07275315

PF-07275315 (Tilrekimig) is a trispecific antibody targeting TSLP, IL-4, and IL-13 currently in phase 2 clinical development for the treatment of asthma (NCT06977581) and atopic dermatitis (NCT05995964). It targets three ligands utilizing a FAb-based modular design (86). Phase 1 studies evaluating the safety and tolerability of IV dosed PF-07275315 have been completed, but results are not yet available. The phase 2 study (NCT06977581) will evaluate the efficacy and safety of SC dosed PF-07275315 in patients with moderate-to-severe asthma on maintenance therapy (ICS/LABA). Primary outcomes of the study will be the change from baseline in FEV1 and safety at week 12.

### IBI3002

IBI3002 is a bispecific antibody targeting TSLP and IL-4RA currently in phase 1 clinical development (NCT06454240). It is a bivalent IgG1-scFv2 fusion antibody with the scFv domains connected to the C-terminal end of the IgG1 Fc region by an optimized linker (87). *In vitro* studies presented at ATS 2025 demonstrate that IBI3002 is similar to a benchmark anti-IL-4RA antibody in inhibition of IL-4/13 binding to IL-4RA and more potent than a benchmark anti-TSLP antibody at inhibiting TSLP binding to TSLPR. Synergistic inhibition of IL-4/IL-13/TSLP-induced TARC expression by PBMCs was observed, with both IBI3002 and a combination of anti-IL-4RA and anti-TSLP demonstrating better inhibition of TARC compared with anti-IL-4RA or anti-TSLP alone. In an *in vivo* model of OVA-induced lung inflammation in IL-4/IL-4R/TSLP/TSLPR humanized mice, IBI3002 treatment resulted in fewer total cell counts in BALF compared with equivalent doses of anti-IL-4RA or anti-TSLP alone. In this model, reductions in eosinophils, neutrophils, and macrophages were similar in IBI3002-treated mice compared with anti-TSLP-treated mice, and serum IgE levels were similar between IBI3002 and anti-IL-4RA-treated mice. The phase 1 study (NCT06454240) is a two-part multiple ascending dose study. In part A, doses of IBI3002 (150–900 mg SC and 600 mg IV) will be evaluated compared with placebo in healthy volunteers, and in part B, two doses of IBI3002 (300 and 900 mg SC) will be evaluated compared with placebo in patients with asthma. Both phases of the study will evaluate safety, tolerability, PK, and immunogenicity.

### ATI-052

ATI-052 is a bispecific antibody targeting TSLP and IL-4R currently in phase 1 clinical development. It leverages the anti-TSLP Fab domains of bosakitug with anti-IL-4R scFv domains linked to the N-terminal end of the VH domain. The Fc domain contains a YTE mutation for half-life extension (64). Preclinical *in vitro* data released by sponsor via corporate presentation demonstrate that ATI-052 is 4× more potent at inhibiting IL-4/TSLP-induced TARC expression by PBMC than a combination of benchmark anti-TSLP and anti-IL-4RA antibodies. Sponsor has reported that a phase 1a/1b program is to initiate that evaluates safety and PK of ATI-052 in SAD and MAD cohorts followed by a proof-of-concept evaluation in patients from up to two undisclosed indications.

### HB0056

HB0056 is a bispecific antibody targeting TSLP and IL-11 currently in phase 1 clinical development (NCT06612970). It is comprised of an anti-TSLP IgG with an anti-IL-11 single-chain nanoantibody linked on the N-terminus of the IgG. IL-11 is a proinflammatory and profibrotic cytokine that has been implicated in the pathogenesis of multiple chronic inflammatory diseases including asthma and COPD (88, 89). While inhibition of IL-11 may reduce inflammation and fibrosis associated with disease pathogenesis, IL-11 also promotes megakaryocytopoiesis, erythropoiesis, thrombopoiesis, and inhibition of proinflammatory cytokine release by macrophages and monocytes, and the overall impact of suppressing IL-11 in patients is unknown. Several companies are developing IL-11 targeted monotherapies. These programs are in phase 1, and clinical safety and efficacy of IL-11 inhibition have not yet been robustly demonstrated. Sponsor has indicated potential future development of HB0056 in respiratory indications including asthma, COPD, and IPF (90).

### APG333 + APG777

APG333 is a monoclonal anti-TSLP antibody currently in phase 1 development (ACTRN12624001358538). It will be developed as part of a combination product alongside APG777, an anti-IL-13 monoclonal antibody. Preclinical *in vitro* studies presented at ATS 2025 demonstrate that the combination of APG333 + APG777 prevents barrier loss induced by TSLP and IL-13 to a greater degree than benchmark anti-IL-4RA or anti-TSLP antibodies alone in a COPD lung epithelial cell model (91). Treatment with APG333 + APG777 inhibited TSLP/IL-33 release of TARC and MDC by PBMCs to a greater degree than comparator monotherapy treatment (anti-IL-13, anti-IL-4RA, or anti-TSLP). Inhibition of TARC was more potent with APG333 + APG777 than with an anti-TSLP/anti-IL-13 multispecific benchmark.

## Preclinical stage multispecific antibodies

Additional bispecific alarmin antibodies are in various stages of preclinical development (Table 3). HXN-1012 is a bispecific antibody targeting TSLP and IL-13. In preclinical *in vitro* assays presented at ATS 2025, HXN-1012 demonstrated more complete inhibition of TARC expression in PBMCs stimulated with TSLP and IL-13 compared with an anti-TSLP/anti-IL-13 multispecific benchmark (92). HXN-1013 is a bispecific antibody targeting TSLP and IL-33 (93). ZW1528 is an asymmetric bispecific antibody targeting IL-33 and IL-4RA with extended half-life in preclinical development (94). Preclinical evaluations presented at ATS 2025 demonstrated that ZW1528 blocks IL-4 and IL-13 binding with similar potency as a benchmark anti-IL-4RA antibody, and IL-33 binding with similar potency to a benchmark anti-IL-33 antibody. Greater inhibition of IL-13/IL-33-induced CCL2 expression in HEK cells was observed with ZW1528 compared with anti-IL-4RA or anti-IL-33 alone.

## Conclusions

Given their redundant functions across these pathways, understanding the *in vivo* source, expression levels, and regulation of tissue-derived signals controlling TSLP, IL-33, and IL-25 production is essential for future studies. A better understanding of the clinical utility of IL-33 and IL-25 is needed, considering the well-established role of anti-TSLP therapeutics in clinical studies. It is important to understand the compensatory mechanism of interplay and the role of these alarmins in fibroblasts and epithelial cells in the context of tissue immunity regulation and remodeling. While the transcription factor framework is a useful starting point, even overlapping signals such as IL-25 and IL-33 likely have non-redundant functions that will need to be carefully dissected. Although data from robust phase 2 clinical studies are needed to establish proof-of-concept for combination therapies targeting alarmins plus an additional target, emerging preclinical data for multiple drug candidates and the phase 1b data demonstrating lunsekimig treatment resulted in deep FeNO reductions are promising.

There are limited data available to date on the safety of combined pathway inhibition with multiple biologic therapies in inflammatory respiratory disease. However, clinical data from case reports (Table 4), as well as data from dupilumab plus itepekimab and from the lunsekimig study in patients with asthma, demonstrate favorable safety and tolerability. Specifically, in a phase 1b study, lunsekimig was well-tolerated. Adverse events were equally distributed in both active and placebo groups; all were mild or moderate in severity. There were no serious adverse events, deaths, or safety concerns identified based on vital signs, electrocardiograms, or laboratory parameters. No infection-related adverse events were reported. However, experience with combined biologic therapy in other chronic inflammatory diseases has been mixed. In rheumatoid arthritis, co-treatment with anti-TNFα and anti-IL1 mAbs and the combination of anti-TNFα mAb and abatacept (CTLA4-Fc/T-cell activation inhibitor) have been evaluated. Neither combination induced enhanced efficacy compared with monotherapy, and were associated with more SAEs (101). In Crohn's disease, a combination of infliximab (anti-TNFα mAb) and natalizumab (anti-α4β1 mAb) resulted in enhanced efficacy with no difference in adverse events between the combination treatment group and those receiving infliximab alone (102). A phase 2 randomized controlled trial evaluating the combination of guselkumab (anti-IL-23 mAb) and golimumab (anti-TNFα mAb) demonstrated significantly improved clinical response and clinical remission compared with monotherapy treatment with no difference between groups in adverse events and serious infections (103).

**Table 4:** Combination therapy clinical reports in asthma.

Biologics used	Patient characteristics	Baseline biomarker	Key outcome	Safety	Reference
Omalizumab (ongoing) + mepolizumab (added)—dose NR	55-years-old female, severe persistent allergic + eosinophilic asthma (case report)	IgE: elevated NR; eosinophils: NR	Marked clinical improvement; prednisone tapered from 20 to 4 mg/day after adding mepolizumab	None reported	Dedaj et al. (95)
Case 1: omalizumab 375 mg Q4W + mepolizumab 100 mg Q4W (concomitant)	Case 1: 65-years-old female with refractory ABPA + asthma	Case 1: IgE 7,699—rose on therapy to 19,032 IU/mL; eosinophils 152 reduced to 0 cells/µL after therapy	Case 1: elimination of monthly exacerbations; FEV1 improved from 1.36 to 1.56 L; corticosteroids withdrawn; radiographic stability at 6 months	Case 1: no adverse effects reported over the reported follow-up	Huang et al. (96)
Case 2: omalizumab + mepolizumab (same dosing as case 1)	Case 2: 72-years-old female with refractory ABPA + asthma	Case 2: baseline IgE and eosinophils reported in paper (high IgE, eosinophilia)—exact values NR	Case 2: steroid-sparing; no exacerbations; follow-up 43 months reported with radiographic stability	Case 2: none reported	
Case 1: dupilumab (ongoing) + mepolizumab or menralizumab or tezepelumab added	Case 1: adults with severe asthma + atopic dermatitis (52-year-old male and 41-years-old male)	Case 1: FeNO 25–32 ppb and eosinophils 600–0 over a 3-year period	Improved control of both asthma and atopic dermatitis	No major AEs reported	Matsumoto et al. (97)
Case 2: omalizumab (ongoing) + dupilumab added		Case 2: FeNO 15–11 ppb and eosinophils 450–350 over a 6-year period			
Various combinations reported (omalizumab + mepolizumab; dupilumab + benralizumab; omalizumab + dupilumab, etc.)—dosing NR for most	Case Series: 10 patients (mixed severe asthma phenotypes; overlapping comorbidities)	Mixed (some high IgE, some high eosinophils)	Among the 10 cases, the majority had clinical benefit including reduced exacerbations and steroid reduction	Short-term safety acceptable in reported cases; longer-term data limited.	Chen et al. (98)
Omalizumab (ongoing) + mepolizumab—some overlapping therapy described in reports	Two patients with severe allergic asthma refractory to multiple therapies	IgE 115 IU/mL	Clinical course improved after adding a second biologic including steroid reduction	NR	Lombardi et al. (99)
Multiple combinations of biologics (anti-IL-5, IL-4, IgE) and different indications.	A total of 25 patients (15 women, 10 men; median age: 54 years). Fifteen patients concomitantly received two biologics approved for asthma: 8 were treated for comorbidities such as CRSwNP, atopic dermatitis, urticaria, or EGPA, while seven received treatment for a combined action on asthma control	NR	The median duration of dual treatment (time point was 9 months (3–38 months) in group A and 24 months (6–49 months) in group B. In group A, the dual treatment was stopped in four patients: in all cases, this was done because of clinical ineffectiveness, not because of adverse effects. All other patients continue to receive two or three biologics concomitantly, with currently no reported adverse effects during this treatment (time point: February 2022)	Treatment well-tolerated	Lommatzsch et al. (100)
Group A received biologics approved for asthma management; group B received one biologic for asthma and another (not approved for asthma)					

EGPA, eosinophilic granulomatosis with polyangiitis; NR, not reported.

As clinical development programs evaluating combined targeting of multiple inflammatory pathways progress, it will be critical to carefully consider the pathways being co-targeted to balance the total immunomodulatory effect of the combination and to thoroughly monitor safety and efficacy to ensure that benefit–risk profiles are commensurate with the severity of disease. Despite the limited number of reported cases, current data provide reassurance that the combination of two well-tolerated biologic therapies for the treatment of asthma may be safe. Overall, these data indicate that blocking an alarmin in combination with a downstream target, thereby selectively inhibiting more than one pathway, could be the next frontier for biologic therapeutics.

## References

[1] Duchesne M Okoye I Lacy P. Epithelial cell alarmin cytokines: frontline mediators of the asthma inflammatory response. Front Immunol. (2022) 13:975914. 10.3389/fimmu.2022.975914 36311787 PMC9616080

[2] Ebina-Shibuya R Leonard WJ. Role of thymic stromal lymphopoietin in allergy and beyond. Nat Rev Immunol. (2023) 23(1):24–37. 10.1038/s41577-022-00735-y 35650271 PMC9157039

[3] Stanbery AG Smita S von Moltke J Wojno ED Ziegler SF. TSLP, IL-33, and IL-25: not just for allergy and helminth infection. J Allergy Clin Immunol. (2022) 150(6):1302–13. 10.1016/j.jaci.2022.07.003 35863509 PMC9742339

[4] Lipworth BJ Han JK Desrosiers M Hopkins C Lee SE Mullol J Tezepelumab in adults with severe chronic rhinosinusitis with nasal polyps. N Engl J Med. (2025) 392(12):1178–88. 10.1056/NEJMoa2414482 40106374

[5] Brusselle GG Koppelman GH. Biologic therapies for severe asthma. N Engl J Med. (2022) 386(2):157–71. 10.1056/NEJMra2032506 35020986

[6] Soumelis V Reche PA Kanzler H Yuan W Edward G Homey B Human epithelial cells trigger dendritic cell–mediated allergic inflammation by producing TSLP. Nat Immunol. (2002) 3(7):673–80. 10.1038/ni805 12055625

[7] Yao W Zhang Y Jabeen R Nguyen ET Wilkes DS Tepper RS Interleukin-9 is required for allergic airway inflammation mediated by the cytokine TSLP. Immunity. (2013) 38(2):360–72. 10.1016/j.immuni.2013.01.007 23376058 PMC3582776

[8] Kim BS Siracusa MC Saenz SA Noti M Monticelli LA Sonnenberg GF TSLP elicits IL-33-independent innate lymphoid cell responses to promote skin inflammation. Sci Transl Med. (2013) 5(170):170ra16. 10.1126/scitranslmed.3005374 23363980 PMC3637661

[9] Roan F Obata-Ninomiya K Ziegler SF. Epithelial cell-derived cytokines: more than just signaling the alarm. J Clin Invest. (2019) 129(4):1441–51. 10.1172/JCI124606 30932910 PMC6436879

[10] Semlali A Jacques E Koussih L Gounni AS Chakir J. Thymic stromal lymphopoietin-induced human asthmatic airway epithelial cell proliferation through an IL-13-dependent pathway. J Allergy Clin Immunol. (2010) 125(4):844–50. 10.1016/j.jaci.2010.01.044 20236697

[11] Allakhverdi Z Comeau MR Jessup HK Yoon BR Brewer A Chartier S Thymic stromal lymphopoietin is released by human epithelial cells in response to microbes, trauma, or inflammation and potently activates mast cells. J Exp Med. (2007) 204(2):253–8. 10.1084/jem.20062211 17242164 PMC2118732

[12] Nagarkar DR Poposki JA Comeau MR Biyasheva A Avila PC Schleimer RP Airway epithelial cells activate TH2 cytokine production in mast cells through IL-1 and thymic stromal lymphopoietin. J Allergy Clin Immunol. (2012) 130(1):225–32.e4. 10.1016/j.jaci.2012.04.019 22633328 PMC3387295

[13] Gieseck RL 3rd Wilson MS Wynn TA. Type 2 immunity in tissue repair and fibrosis. Nat Rev Immunol. (2018) 18(1):62–76. 10.1038/nri.2017.90 28853443

[14] Hsieh YA Hsiao YH Ko HK Shen YL Huang CW Perng DW House dust mites stimulate thymic stromal lymphopoietin production in human bronchial epithelial cells and promote airway remodeling through activation of PAR2 and ERK signaling pathway. Sci Rep. (2024) 14(1):28649. 10.1038/s41598-024-79226-0 39562597 PMC11577110

[15] Dunn JLM Shoda T Caldwell JM Wen T Aceves SS Collins MH Esophageal type 2 cytokine expression heterogeneity in eosinophilic esophagitis in a multisite cohort. J Allergy Clin Immunol. (2020) 145(6):1629–40.e4. 10.1016/j.jaci.2020.01.051 32197970 PMC7309223

[16] Fornasa G Tsilingiri K Caprioli F Botti F Mapelli M Meller S Dichotomy of short and long thymic stromal lymphopoietin isoforms in inflammatory disorders of the bowel and skin. J Allergy Clin Immunol. (2015) 136(2):413–22. 10.1016/j.jaci.2015.04.011 26014813 PMC4534776

[17] Verstraete K Peelman F Braun H Lopez J Van Rompaey D Dansercoer A Structure and antagonism of the receptor complex mediated by human TSLP in allergy and asthma. Nat Commun. (2017) 8:14937. 10.1038/ncomms14937 28368013 PMC5382266

[18] Howell I Howell A Pavord ID. Type 2 inflammation and biological therapies in asthma: targeted medicine taking flight. J Exp Med. (2023) 220(7):e20221212. 10.1084/jem.20221212 37265457 PMC10239209

[19] Porsbjerg C Melen E Lehtimaki L Shaw D. Asthma. Lancet. (2023) 401(10379):858–73. 10.1016/S0140-6736(22)02125-0 36682372

[20] Calderon AA Dimond C Choy DF Pappu R Grimbaldeston MA Mohan D Targeting interleukin-33 and thymic stromal lymphopoietin pathways for novel pulmonary therapeutics in asthma and COPD. Eur Respir Rev. (2023) 32(167):220144. 10.1183/16000617.0144-2022 36697211 PMC9879340

[21] Torgerson DG Ampleford EJ Chiu GY Gauderman WJ Gignoux CR Graves PE Meta-analysis of genome-wide association studies of asthma in ethnically diverse North American populations. Nat Genet. (2011) 43(9):887–92. 10.1038/ng.888 21804549 PMC3445408

[22] Kottyan LC Parameswaran S Weirauch MT Rothenberg ME Martin LJ. The genetic etiology of eosinophilic esophagitis. J Allergy Clin Immunol. (2020) 145(1):9–15. 10.1016/j.jaci.2019.11.013 31910986 PMC6984394

[23] Andreasson LM Dyhre-Petersen N Hvidtfeldt M Jorgensen GO Von Bulow A Klein DK Airway hyperresponsiveness correlates with airway TSLP in asthma independent of eosinophilic inflammation. J Allergy Clin Immunol. (2024) 153(4):988–97.e11. 10.1016/j.jaci.2023.11.915 38081546

[24] Ying S O'Connor B Ratoff J Meng Q Fang C Cousins D Expression and cellular provenance of thymic stromal lymphopoietin and chemokines in patients with severe asthma and chronic obstructive pulmonary disease. J Immunol. (2008) 181(4):2790–8. 10.4049/jimmunol.181.4.2790 18684970

[25] Menzies-Gow A Corren J Bourdin A Chupp G Israel E Wechsler ME Tezepelumab in adults and adolescents with severe, uncontrolled asthma. N Engl J Med. (2021) 384(19):1800–9. 10.1056/NEJMoa2034975 33979488

[26] Lefrancais E Cayrol C. Mechanisms of IL-33 processing and secretion: differences and similarities between IL-1 family members. Eur Cytokine Netw. (2012) 23(4):120–7. 10.1684/ecn.2012.0320 23306193

[27] Cayrol C Girard JP. Interleukin-33 (IL-33): a nuclear cytokine from the IL-1 family. Immunol Rev. (2018) 281(1):154–68. 10.1111/imr.12619 29247993

[28] Travers J Rochman M Miracle CE Habel JE Brusilovsky M Caldwell JM Chromatin regulates IL-33 release and extracellular cytokine activity. Nat Commun. (2018) 9(1):3244. 10.1038/s41467-018-05485-x 30108214 PMC6092330

[29] Hung LY Tanaka Y Herbine K Pastore C Singh B Ferguson A Cellular context of IL-33 expression dictates impact on anti-helminth immunity. Sci Immunol. (2020) 5(53):eabc6259. 10.1126/sciimmunol.abc6259 33188058 PMC8257082

[30] Luthi AU Cullen SP McNeela EA Duriez PJ Afonina IS Sheridan C Suppression of interleukin-33 bioactivity through proteolysis by apoptotic caspases. Immunity. (2009) 31(1):84–98. 10.1016/j.immuni.2009.05.007 19559631

[31] Palmer G Gabay C. Interleukin-33 biology with potential insights into human diseases. Nat Rev Rheumatol. (2011) 7(6):321–9. 10.1038/nrrheum.2011.53 21519352

[32] Cohen ES Scott IC Majithiya JB Rapley L Kemp BP England E Oxidation of the alarmin IL-33 regulates ST2-dependent inflammation. Nat Commun. (2015) 6:8327. 10.1038/ncomms9327 26365875 PMC4579851

[33] England E Rees DG Scott IC Carmen S Chan DTY Chaillan Huntington CE Tozorakimab (MEDI3506): an anti-IL-33 antibody that inhibits IL-33 signalling via ST2 and RAGE/EGFR to reduce inflammation and epithelial dysfunction. Sci Rep. (2023) 13(1):9825. 10.1038/s41598-023-36642-y 37330528 PMC10276851

[34] Liu X Li M Wu Y Zhou Y Zeng L Huang T. Anti-IL-33 antibody treatment inhibits airway inflammation in a murine model of allergic asthma. Biochem Biophys Res Commun. (2009) 386(1):181–5. 10.1016/j.bbrc.2009.06.008 19508862

[35] Kim YH Park CS Lim DH Ahn SH Son BK Kim JH Beneficial effect of anti-interleukin-33 on the murine model of allergic inflammation of the lower airway. J Asthma. (2012) 49(7):738–43. 10.3109/02770903.2012.702841 22799279

[36] Lee HY Rhee CK Kang JY Byun JH Choi JY Kim SJ Blockade of IL-33/ST2 ameliorates airway inflammation in a murine model of allergic asthma. Exp Lung Res. (2014) 40(2):66–76. 10.3109/01902148.2013.870261 24446582

[37] Allinne J Scott G Lim WK Birchard D Erjefalt JS Sanden C IL-33 blockade affects mediators of persistence and exacerbation in a model of chronic airway inflammation. J Allergy Clin Immunol. (2019) 144(6):1624–37.e10. 10.1016/j.jaci.2019.08.039 31562870

[38] Zoltowska AM Lei Y Fuchs B Rask C Adner M Nilsson GP. The interleukin-33 receptor ST2 is important for the development of peripheral airway hyperresponsiveness and inflammation in a house dust mite mouse model of asthma. Clin Exp Allergy. (2016) 46(3):479–90. 10.1111/cea.12683 26609909

[39] Verma M Liu S Michalec L Sripada A Gorska MM Alam R. Experimental asthma persists in IL-33 receptor knockout mice because of the emergence of thymic stromal lymphopoietin-driven IL-9(+) and IL-13(+) type 2 innate lymphoid cell subpopulations. J Allergy Clin Immunol. (2018) 142(3):793–803.e8. 10.1016/j.jaci.2017.10.020 29132961 PMC5945345

[40] Choi Y Kim YM Lee HR Mun J Sim S Lee DH Eosinophil extracellular traps activate type 2 innate lymphoid cells through stimulating airway epithelium in severe asthma. Allergy. (2020) 75(1):95–103. 10.1111/all.13997 31330043

[41] Hong JY Bentley JK Chung Y Lei J Steenrod JM Chen Q Neonatal rhinovirus induces mucous metaplasia and airways hyperresponsiveness through IL-25 and type 2 innate lymphoid cells. J Allergy Clin Immunol. (2014) 134(2):429–39. 10.1016/j.jaci.2014.04.020 24910174 PMC4119851

[42] Xu M Dong C. IL-25 in allergic inflammation. Immunol Rev. (2017) 278(1):185–91. 10.1111/imr.12558 28658555

[43] Borowczyk J Shutova M Brembilla NC Boehncke WH. IL-25 (IL-17E) in epithelial immunology and pathophysiology. J Allergy Clin Immunol. (2021) 148(1):40–52. 10.1016/j.jaci.2020.12.628 33485651

[44] Ballantyne SJ Barlow JL Jolin HE Nath P Williams AS Chung KF Blocking IL-25 prevents airway hyperresponsiveness in allergic asthma. J Allergy Clin Immunol. (2007) 120(6):1324–31. 10.1016/j.jaci.2007.07.051 17889290

[45] Gregory LG Jones CP Walker SA Sawant D Gowers KH Campbell GA IL-25 drives remodelling in allergic airways disease induced by house dust mite. Thorax. (2013) 68(1):82–90. 10.1136/thoraxjnl-2012-202003 23093652 PMC3534261

[46] Patel NN Kohanski MA Maina IW Workman AD Herbert DR Cohen NA. Sentinels at the wall: epithelial-derived cytokines serve as triggers of upper airway type 2 inflammation. Int Forum Allergy Rhinol. (2019) 9(1):93–9. 10.1002/alr.22206 30260580 PMC6318004

[47] Barlow JL Peel S Fox J Panova V Hardman CS Camelo A IL-33 is more potent than IL-25 in provoking IL-13-producing nuocytes (type 2 innate lymphoid cells) and airway contraction. J Allergy Clin Immunol. (2013) 132(4):933–41. 10.1016/j.jaci.2013.05.012 23810766

[48] Nakanishi W Yamaguchi S Matsuda A Suzukawa M Shibui A Nambu A IL-33, but not IL-25, is crucial for the development of house dust mite antigen-induced allergic rhinitis. PLoS One. (2013) 8(10):e78099. 10.1371/journal.pone.0078099 24205109 PMC3808342

[49] Beale J Jayaraman A Jackson DJ Macintyre JDR Edwards MR Walton RP Rhinovirus-induced IL-25 in asthma exacerbation drives type 2 immunity and allergic pulmonary inflammation. Sci Transl Med. (2014) 6(256):256ra134. 10.1126/scitranslmed.3009124 25273095 PMC4246061

[50] TEZSPIRE. Prescribing information. AstraZeneca (2023). Available online at: https://www.azpicentral.com/pi.html?product=tezspire (Accessed August 24, 2025).

[51] Corren J Parnes JR Wang L Mo M Roseti SL Griffiths JM Tezepelumab in adults with uncontrolled asthma. N Engl J Med. (2017) 377(10):936–46. 10.1056/NEJMoa1704064 28877011

[52] Singh D Brightling CE Rabe KF Han MK Christenson SA Drummond MB Efficacy and safety of tezepelumab versus placebo in adults with moderate to very severe chronic obstructive pulmonary disease (COURSE): a randomised, placebo-controlled, phase 2a trial. Lancet Respir Med. (2025) 13(1):47–58. 10.1016/S2213-2600(24)00324-2 39653044

[53] Rabe KF Celli BR Wechsler ME Abdulai RM Luo X Boomsma MM Safety and efficacy of itepekimab in patients with moderate-to-severe COPD: a genetic association study and randomised, double-blind, phase 2a trial. Lancet Respir Med. (2021) 9(11):1288–98. 10.1016/S2213-2600(21)00167-3 34302758

[54] Sanofi. Press Release: itepekimab met the primary endpoint in one of two COPD phase 3 studies [press release]. (2025).

[55] Singh D Guller P Reid F Doffman S Seppala U Psallidas I A phase 2a trial of the IL-33 monoclonal antibody tozorakimab in patients with COPD: FRONTIER-4. Eur Respir J. (2025) 66(1):2402231. 10.1183/13993003.02231-2024 40154559 PMC12256803

[56] Yousuf AJ Mohammed S Carr L Yavari Ramsheh M Micieli C Mistry V Astegolimab, an anti-ST2, in chronic obstructive pulmonary disease (COPD-ST2OP): a phase 2a, placebo-controlled trial. Lancet Respir Med. (2022) 10(5):469–77. 10.1016/S2213-2600(21)00556-7 35339234

[57] Genentech. Genentech provides update on astegolimab in chronic obstructive pulmonary disease [press release]. (2025).

[58] Upstream Corporate Presentation. Upstream bio (2025). Available online at: https://investors.upstreambio.com/static-files/7d692e7b-6244-4a51-9248-d85f48e78007 (Accessed September 2, 2025).

[59] Corren J Pham TH Garcia Gil E Salapa K Ren P Parnes JR Baseline type 2 biomarker levels and response to tezepelumab in severe asthma. Allergy. (2022) 77(6):1786–96. 10.1111/all.15197 34913186 PMC9306691

[60] Fei Y Li N Qian W Fan Y Shen Y Wang Q A phase 1, randomized, double-blind, placebo-controlled, dose escalation study to evaluate the safety, tolerability, pharmacokinetics and immunogenicity of SHR-1905, a long-acting anti-thymic stromal lymphopoietin antibody, in healthy subjects. Front Pharmacol. (2024) 15:1400696. 10.3389/fphar.2024.1400696 39076593 PMC11284144

[61] Chen Q Yu C Zou Y Ye L Yang J Xiang Z A phase 1 study of SHR-1905, a long-acting anti-TSLP monoclonal antibody, in healthy subjects and patients with asthma. Eur Respir J. (2024) 64(suppl 68):OA1972. 10.1183/13993003.congress-2024.OA1972

[62] Numazaki M Abe M Hanaoka K Imamura E Maeda M Kimura A ASP7266, a novel antibody against human thymic stromal lymphopoietin receptor for the treatment of allergic diseases. J Pharmacol Exp Ther. (2022) 380(1):26–33. 10.1124/jpet.121.000686 34728559

[63] Singh D Deykin A Lloyd P Nestorov I Kalra A Biswas S A multiple ascending-dose study with verekitug, a novel antibody to the human thymic stromal lymphopoietin receptor, in adults with asthma. Am J Respir Crit Care Med. (2024) 209:A6996. 10.1164/ajrccm-conference.2024.209.1_MeetingAbstracts.A6996

[64] Aclaris corporate presentation. Aclaris (2025). Available online at: https://investor.aclaristx.com/static-files/e0078ebd-0054-4ef2-ab2e-a8bdeb72356d (Accessed July 24, 2025).

[65] Tian X Liu R Zhang L Qi L Xue W Wang S Safety, tolerability, pharmacokinetics, and immunogenicity of a human monoclonal antibody TQC2731 targeting thymic stromal lymphopoietin (TSLP) in healthy Chinese adults. Eur Respir J. (2024) 64(suppl 68):PA4860. 10.1164/ajrccm.2025.211.Abstracts.A3424

[66] Di Y Yang L Zhou J Zhang L Huang Y Jia Y Translational investigation of CM326 from preclinical studies to randomized phase I clinical trials in healthy adults. BioDrugs. (2025) 39(3):487–98. 10.1007/s40259-025-00714-4 40185989

[67] Shi L Yu M Jin Y Chen P Mu G Tam SH A novel monoclonal antibody against human thymic stromal lymphopoietin for the treatment of TSLP-mediated diseases. Front Immunol. (2024) 15:1442588. 10.3389/fimmu.2024.1442588 39726595 PMC11670205

[68] Han C Fung I Zhang D Jin Y Chen P Tam S Phase 1 safety and pharmacokinetics study of TAVO101, an anti-human thymic stromal lymphopoietin antibody for the treatment of allergic inflammatory conditions. J Clin Pharmacol. (2024) 65:28–40. 10.1002/jcph.6115 39141432

[69] Komori H Lore M Postlethwaite H Manga V Wittmer L Ortega H. A differentiated anti-TSLP antibody, provides distinct epitope binding profile and superior potency compared to tezepelumab. Am J Respir Crit Care Med. (2025) 211:A1393. 10.1164/ajrccm.2025.211.Abstracts.A1393

[70] Doffman S Dosanjh D Sadiq M Asimus S Cooper J Zhou X-H Phase 1 safety and efficacy of AZD8630/AMG 104 inhaled anti-TSLP in healthy volunteers and patients with asthma on medium-high dose inhaled corticosteroid (ICS) and long-acting beta-agonist (LABA) with elevated baseline fractional exhaled nitric oxide (FeNO) [abstract]. Am J Respir Crit Care Med. (2024) 209:A1386. 10.1164/ajrccm-conference.2024.209.1_MeetingAbstracts.A1386

[71] Asimus S Sadiq WM Cooper J Dosanjh D Zhou X-H Pandya H Pharmacokinetics of AZD8630/AMG 104 inhaled anti-TSLP in healthy adults and asthma patients. Eur Respir J. (2024) 64(suppl 68):PA3558. 10.1183/13993003.congress-2024.PA3558

[72] O'Byrne PM Panettieri RA Jr Taube C Brindicci C Fleming M Altman P. Development of an inhaled anti-TSLP therapy for asthma. Pulm Pharmacol Ther. (2023) 78:102184. 10.1016/j.pupt.2022.102184 36535465

[73] Gauvreau GM Hohlfeld JM FitzGerald JM Boulet LP Cockcroft DW Davis BE Inhaled anti-TSLP antibody fragment, ecleralimab, blocks responses to allergen in mild asthma. Eur Respir J. (2023) 61(3):2201193. 10.1183/13993003.01193-2022 36822634 PMC9996823

[74] Gawde T Mayawala K Aliprantis A Escalante JH Han H Ullas S A long-acting high affinity anti-TSLP antibody (GB-0895) for severe asthma identified leveraging a proprietary machine learning platform. Eur Respir J. (2024) 64(suppl 68):PA2983. 10.1183/13993003.congress-2024.PA2983

[75] Ran H Liu D Zhou Y Dong L Dong L Wang Y AI-guided generation and development of HXN-1011, a highly potent anti-TSLP biparatopic antibody. Am J Respir Crit Care Med. (2025) 211:A7443. 10.1164/ajrccm.2025.211.Abstracts.A7443

[76] Wechsler ME Ruddy MK Pavord ID Israel E Rabe KF Ford LB Efficacy and safety of itepekimab in patients with moderate-to-severe asthma. N Engl J Med. (2021) 385(18):1656–68. 10.1056/NEJMoa2024257 34706171

[77] Tian X Liu R Zhang L Qi L Xue W Wang S Safety, tolerability, pharmacokinetics, and immunogenicity of a human monoclonal antibody TQC2938 targeting the IL-33/ST2 axis in healthy Chinese adults. Am J Respir Crit Care Med. (2025) 211:A3425. 10.1164/ajrccm.2025.211.Abstracts.A3425

[78] Zhang H Zheng W Peng R Wu D Hu Y Sun T First-in-human study on tolerability, pharmacokinetics and pharmacodynamics of single and multiple escalating doses of XKH001, a recombinant humanized monoclonal antibody against IL-25 in healthy Chinese volunteers. Expert Opin Invest Drugs. (2025) 34(1-2):81–7. 10.1080/13543784.2025.2453162 39815604

[79] Xu G Paglialunga S Qian X Ding R Webster K van Haarst A Evaluation of the safety, tolerability, pharmacokinetics and pharmacodynamics of SM17 in healthy volunteers: results from pre-clinical models and a first-in-human, randomized, double blinded clinical trial. Front Immunol. (2024) 15:1495540. 10.3389/fimmu.2024.1495540 39717777 PMC11663749

[80] DUPIXENT. Prescribing information. Regeneron (2025). Available online at: https://www.regeneron.com/downloads/dupixent_fpi.pdf (Accessed August 24, 2025).

[81] NUCALA. Prescribing information. GSK (2025). Available online at: https://gskpro.com/content/dam/global/hcpportal/en_US/Prescribing_Information/Nucala/pdf/NUCALA-PI-PIL-IFU-COMBINED.PDF (Accessed August 24, 2025).

[82] FASENRA. Prescribing Information. AstraZeneca (2025). Available online at: https://www.azpicentral.com/pi.html?product=fasenra (Accessed August 24, 2025).

[83] Deiteren A Bontinck L Conickx G Vigan M Dervaux N Gassiot M A first-in-human, single and multiple dose study of lunsekimig, a novel anti-TSLP/anti-IL-13 NANOBODY(R) compound, in healthy volunteers. Clin Transl Sci. (2024) 17(6):e13864. 10.1111/cts.13864 38924698 PMC11196376

[84] Toh WH Louber J Mahmoud IS Chia J Bass GT Dower SK FcRn mediates fast recycling of endocytosed albumin and IgG from early macropinosomes in primary macrophages. J Cell Sci. (2019) 133(5):jcs235416. 10.1242/jcs.235416 31444284

[85] Deiteren A Krupka E Bontinck L Imberdis K Conickx G Bas S A proof-of-mechanism trial in asthma with lunsekimig, a bispecific NANOBODY molecule. Eur Respir J. (2025) 65(4):2401461. 10.1183/13993003.01461-2024 39884759 PMC12018761

[86] Wynn TA. In pursuit of transformational efficacy for autoimmune, inflammatory, and fibrotic diseases with multi-functional therapeutics [oral presentation]. In: Immunology 2024; Chicago, IL, USA (2024).

[87] Xiong Y Li L Zhou S Tang K He F Gao Y Preclinical characterization of IBI3002, an anti-IL-4R*α* and anti-TSLP bispecific antibody that potently dampens inflammatory response and alleviates asthma in mice. Am J Respir Crit Care Med. (2025) 211:A7432. 10.1164/ajrccm.2025.211.Abstracts.A7432

[88] Fung KY Louis C Metcalfe RD Kosasih CC Wicks IP Griffin MDW Emerging roles for IL-11 in inflammatory diseases. Cytokine. (2022) 149:155750. 10.1016/j.cyto.2021.155750 34689057

[89] Kortekaas RK Burgess JK van Orsoy R Lamb D Webster M Gosens R. Therapeutic targeting of IL-11 for chronic lung disease. Trends Pharmacol Sci. (2021) 42(5):354–66. 10.1016/j.tips.2021.01.007 33612289

[90] HuaboBio pipeline. Huabo (2025). Available online at: https://www.huabobio.com/en/product.html (Accessed August 27, 2025).

[91] Barth K Wickman G Barker K Dillinger L. The combination of APG777 (anti-IL-13) and APG333 (anti-TSLP) inhibits central and local drivers of obstructive airway disease. Am J Respir Crit Care Med. (2025) 211:A2489. 10.1164/ajrccm.2025.211.Abstracts.A2489

[92] Ran H Huang J Li H Dong L Liu D Su C AI-guided engineering and generation of bispecific antibodies targeting both IL13 and TSLP for chronic respiratory diseases. Am J Respir Crit Care Med. (2025) 211:A1361. 10.1164/ajrccm.2025.211.Abstracts.A1361

[93] Ran H Huang J Li H Dong L Liu D Zhou Y Bispecific antibody targeting both IL33 and TSLP for asthma and COPD. Am J Respir Crit Care Med. (2025) 211:A1382. 10.1164/ajrccm.2025.211.Abstracts.A1382

[94] Poffenberger M Bhojane P Hardman B Wu B Li J Patel K ZW1528, a bispecific antibody targeting IL-4R*α* and IL-33, potently inhibits key mediators of airway inflammation. Am J Respir Crit Care Med. (2025) 211:A3446. 10.1164/ajrccm.2025.211.Abstracts.A3446

[95] Dedaj R Unsel L. Case study: a combination of mepolizumab and omaluzimab injections for severe asthma. J Asthma. (2019) 56(5):473–4. 10.1080/02770903.2018.1471706 29733738

[96] Huang IH Dang KN Kashyap S Clair NS Sweidan AJ. Duo biologic therapy using mepolizumab and omalizumab in refractory ABPA: two cases. Allergy Asthma Clin Immunol. (2025) 21(1):39. 10.1186/s13223-025-00985-0 40898293 PMC12403339

[97] Matsumoto T Sakurai Y Tashima N Matoba T Kaneko A Fujiki T Dual biologics for severe asthma and atopic dermatitis: synopsis of two cases and literature review. Respirol Case Rep. (2024) 12(1):e01266. 10.1002/rcr2.1266 38074921 PMC10701292

[98] Chen Y Wang L Xie J. Combining dual biologics therapy for severe asthma: a series of ten cases. J Asthma Allergy. (2025) 18:507–17. 10.2147/JAA.S507008 40206518 PMC11980939

[99] Lombardi C Menzella F Passalacqua G. Long-term responsiveness to mepolizumab after failure of omalizumab and bronchial thermoplasty: two triple-switch case reports. Respir Med Case Rep. (2020) 29:100967. 10.1016/j.rmcr.2019.100967 31799113 PMC6881682

[100] Lommatzsch M Suhling H Korn S Bergmann KC Schreiber J Bahmer T Safety of combining biologics in severe asthma: asthma-related and unrelated combinations. Allergy. (2022) 77(9):2839–43. 10.1111/all.15379 35585763

[101] Mutlu MY Tascilar K Schett G. Rationale, current state and opportunities in combining biologic disease modifying antirheumatic drugs in rheumatoid and psoriatic arthritis. Joint Bone Spine. (2023) 90(5):105578. 10.1016/j.jbspin.2023.105578 37076093

[102] Sands BE Kozarek R Spainhour J Barish CF Becker S Goldberg L Safety and tolerability of concurrent natalizumab treatment for patients with Crohn’s disease not in remission while receiving infliximab. Inflamm Bowel Dis. (2007) 13(1):2–11. 10.1002/ibd.20014 17206633

[103] Feagan BG Sands BE Sandborn WJ Germinaro M Vetter M Shao J Guselkumab plus golimumab combination therapy versus guselkumab or golimumab monotherapy in patients with ulcerative colitis (VEGA): a randomised, double-blind, controlled, phase 2, proof-of-concept trial. Lancet Gastroenterol Hepatol. (2023) 8(4):307–20. 10.1016/S2468-1253(22)00427-7 36738762

